# Advanced Biomaterial Delivery of Hypoxia‐Conditioned Extracellular Vesicles (EVs) as a Therapeutic Platform for Traumatic Brain Injury

**DOI:** 10.1002/advs.202504147

**Published:** 2025-09-08

**Authors:** Joshua B. Stein, Songzi Zhang, Eun Ji Roh, Jeffrey Luo, Meizi Chen, Hyunjun Jang, Li Ling Goldston, Brandon Conklin, Inbo Han, Ki‐Bum Lee

**Affiliations:** ^1^ Department of Chemistry and Chemical Biology Rutgers The State University of New Jersey Piscataway NJ 08854 USA; ^2^ Department of Neurosurgery CHA University School of Medicine CHA Bundang Medical Center 59 Yaptap‐ro, Bundang‐gu Seongnam‐si Gyeonggi‐do 13496 Republic of Korea

**Keywords:** angiogenesis, bioorthogonal hydrogel (BIOGEL), extracellular vesicles (EVs), human induced pluripotent stem cell neural progenitor cell (hiPSC‐NPC), hypoxia conditioning, neural repair, neurogenesis, traumatic brain injury (TBI)

## Abstract

Traumatic Brain Injury (TBI) is a common and debilitating injury, causing long‐lasting neurological deficits. Current therapeies for recovery remain inadequate, undersing the urgent need for innovative interventions. In this study, a novel therapeutic approach is introduced that delivers extracellular vesicles (EVs) derived from human‐induced pluripotent stem cell‐derived neural progenitor cells (hiPSC‐NPCs) with a gelatin‐based injectable bioorthogonal hydrogel (BIOGEL). The hiPSC‐NPCs are conditioned with deferoxamine (DFO) to simulate hypoxia, resulting in EVs enriched with neurotrophic and angiogenic factors critical for neural repair. The biomimetic mechanical properties of BIOGEL, similar to those of native brain tissue, contribute to sustained EV delivery and promote neural regeneration. BIOGEL with hypoxia‐conditioned EVs showed significant tissue regeneration in vivo using a rat model of TBI. Our nanomaterial platform reduced cortical lesions, improved neurological and motor recovery, enhanced hippocampal neurogenesis and myelination, and reduced neuroinflammation, demonstrating strong therapeutic potential for neural repair. In summary, this study demonstrated proof‐of‐concept for a multifaceted therapeutic platform that simultaneously targets key pathological features of TBI, providing a scalable and clinically translatable approach to effective neural tissue regeneration. The synergistic combination of hypoxia‐conditioned EVs and biomaterial delivery offers a promising strategy for advancing regenerative medicine techniques for neural repair.

## Graphical abstract

This research introduces a novel approach to enhance neuroregeneration following Traumatic Brain Injury (TBI). Extracellular Vesicles (EVs) are isolated from human neural progenitor cells under hypoxic conditions, leading to enhanced expression of neurogenic and angiogenic factors. Substantial reversal of injury pathology is observed, including increased neurogenesis, angiogenesis, NSC differentiation into neurons, and reduced inflammation.

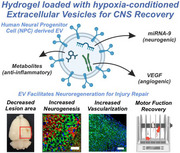

## 1 Introduction

**Figure 1:**
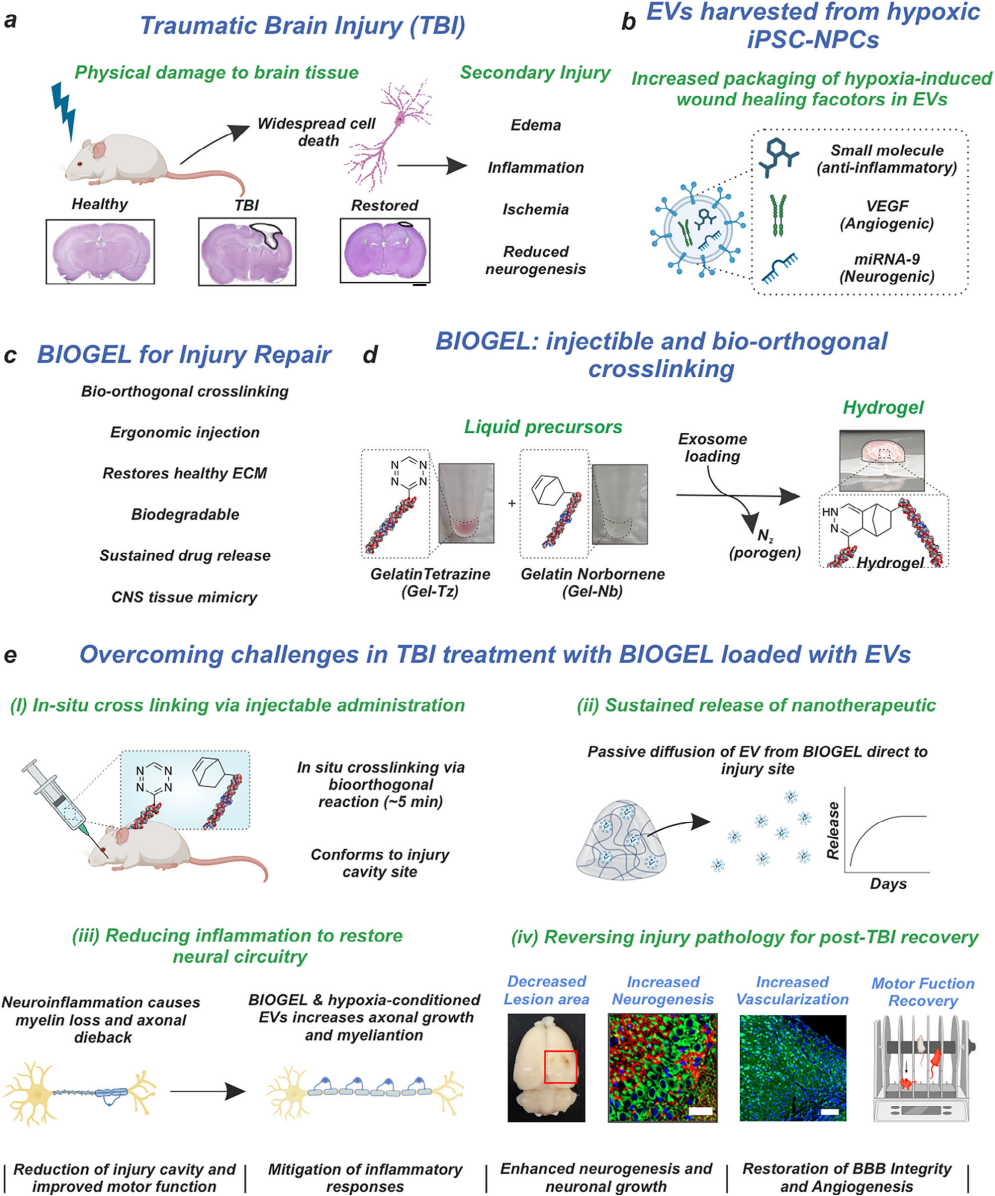
BIOGEL (gelatin‐based bio‐injectable hydrogel) loaded with extracellular vesicles (EVs) produced under hypoxic conditions from human Neural Progenitor Cells (NPCs) aids injury repair following TBI. a) Physical injury to the brain can damage brain tissue and cause neuroinflammation. b) Neural Progenitor Cells (NPCs) were incubated with deferoxamine (DFO) to induce hypoxia and upregulate angiogenic and neurogenic factors. Extracellular vesicles (EVs), with the wound healing factors, were isolated from NPC media. c) We utilized our bio‐orthogonal bio‐injectable gelatin‐based hydrogel for injury repair. d) BIOGEL is comprised of gelatin tetrazine and gelatin norbornene liquid precursors that, when mixed, bio‐orthogonally click and form the hydrogel. e) DFO‐conditioned EVs were administered to mice suffering from Traumatic Brain Injury (TBI) using BIOGEL to promote neurogenesis and injury recovery. The hydrogel addressed the limitations of many therapeutics for TBI for enhanced injury repair.

Traumatic brain injury (TBI) consists of multifaceted pathological processes that frequently result in lasting neurological deficits with limited therapeutic interventions. While primary mechanical damage occurs at impact, a subsequent secondary injury phase initiates neuroinflammatory cascades that drive underlying degeneration and may persist chronically post‐trauma (**Figure** **1**a).^[^ ^1^ ^]^ TBI significantly alters the brain microenvironment, including inflammatory mediator release, reactive gliosis, neuronal death, and oxidative stress. These pathological processes create a hostile milieu that impedes endogenous repair mechanisms and disrupts neural circuit integrity, and efforts have increasingly focused on targeting secondary injury mechanisms.^[^ ^2^ ^]^ However, current therapeutic approaches fail to address the complex pathophysiology, necessitating innovative therapies that target multiple aspects of secondary injury while promoting neural regeneration.^[^ ^3^ ^]^ Given that TBI ranks among the leading causes of mortality and long‐term disability worldwide, this unmet clinical need underscores the importance of developing novel interventions that can effectively modulate the post‐injury microenvironment and enhance endogenous repair mechanisms.^[^ ^4^ ^]^

To this end, stem cell‐based therapies hold promise for Central Nervous System (CNS) regeneration, leveraging their ability to differentiate into neural cells.^[^ ^5^ ^]^ In TBI, where regeneration is impaired, stem cells enhance repair via paracrine signaling, releasing trophic factors to modulate inflammation and promote regeneration.^[^ ^6^ ^]^ However, the clinical translation of stem cell therapies faces substantial challenges due to numerous technical and engineering limitations.^[^ ^7^ ^]^ In response, attention has shifted toward allogeneic approaches, with extracellular vesicles (EVs) emerging as a promising therapeutic modality.^[^ ^8^ ^]^ These endogenous lipid nanoparticles serve as delivery vehicles, mediating intercellular communication through targeted transport of regulatory biomolecules such as gene‐silencing miRNAs, receptor‐activating proteins, and metabolic intermediates—collectively maintaining tissue homeostasis through paracrine signaling networks (Figure 1b).^[^ ^9^ ^]^ EVs have demonstrated multiple regenerative capabilities: they modulate immune system activity, provide neuroprotection, promote neurogenesis, and stimulate angiogenesis.^[^ ^10^ ^]^ However, their therapeutic efficacy inherently depends on both the phenotype of their parent cells and the pathophysiological characteristics of the target tissue microenvironment.^[^ ^11^ ^]^ Despite their considerable therapeutic potential, the clinical translation of EVs is currently limited by several major challenges, including premature cargo release prior to reaching target tissues and off‐target delivery to non‐injured sites. Together, these limitations collectively reduce their in vivo efficacy.^[^ ^12^ ^]^ Consequently, there is an urgent need for advanced delivery systems to improve EV retention and enable sustained therapeutic effects at the injury site.

Advanced hydrogel‐based drug delivery systems (DDSs) can provide a compelling solution for optimizing EV therapeutics, offering controlled delivery to the injury microenvironment and sustained release of nanotherapeutic cargo (Figure 1d). Beyond drug delivery, hydrogels can actively modulate the post‐TBI extracellular matrix (ECM) by providing a supportive scaffold for tissue regeneration, reducing excessive collagen deposition, or sequestering inflammatory molecules (Figure 1d).^[^ ^13^ ^]^ Gelatin‐based hydrogels have gained attention as attractive biomaterial candidates due to their ability to mimic key structural and functional properties of the native extracellular matrix (ECM).^[^ ^14^ ^]^ These hydrogels also promote essential regenerative processes, such as angiogenesis, neovascularization, and blood‐brain barrier (BBB) restoration, highlighting their potential for therapeutic applications in CNS injuries.^[^ ^14^ ^]^ Integrating gelatin‐based nanomaterials with therapeutic EVs offers a sophisticated strategy for neural tissue engineering. This approach can address ECM disruption, promote neuronal regeneration, and mitigate inflammatory processes, including reactive gliosis.

To address these therapeutic challenges in TBI treatment, we developed an advanced hydrogel‐based delivery system for therapeutic EVs (Figure 1a). EVs are derived from human‐induced pluripotent stem cell‐derived neural progenitor cells (hiPSC‐NPCs) cultured under hypoxic conditions to mirror the injury microenvironment. We employed deferoxamine (DFO) to simulate hypoxia, which enhanced the expression of key wound‐healing mediators, particularly miRNA‐9 (a critical neurogenesis regulator) and vascular endothelial growth factor (VEGF)^[^ ^15^ ^]^ (thus addressing ischemia while promoting angiogenesis and BBB restoration) (Figure 1b). The selection of hiPSC‐NPCs was due to their ability to secrete factors with neurotrophic, anti‐inflammatory, and angiogenic properties. These secreted factors are known to support and enhance regenerative processes in endogenous neural stem cells (NSCs) and endothelial cells.^[^ ^16^ ^]^ The isolated EVs were encapsulated within our previously developed injectable, bioorthogonal hydrogel (BIOGEL) (Figure 1c). BIOGEL comprises gelatin‐tetrazine and gelatin‐norbornene liquid precursors that undergo bioorthogonal crosslinking to form a hydrogel matching the mechanical properties of native brain tissue (Figure 1d).^[^ ^17^ ^]^ BIOGEL's injectability allows for minimally invasive administration while facilitating the hydrogel to conform to the injury site. This integrated platform enables both targeted delivery and sustained release of EVs at the injury site, optimizing their therapeutic potential.

Our advanced biomaterial‐based DDS and therapeutic approach showed therapeutic effects through the complementary actions of BIOGEL and EVs when investigated using a rat model of TBI. Substantial therapeutic improvements were observed in vivo, marked by an elevated expression of neuronal markers, robust remyelination, and restored endothelial integrity. These structural and functional enhancements correlated with measurable gains in motor function, as confirmed by histological and behavioral assessments. Increased endogenous levels of brain‐derived neurotrophic factor (BDNF) and neurotrophin receptor p75NTR were found, supporting neural circuit reconstruction^[^ ^18^ ^]^ (Figure 1e). These findings provide novel and compelling proof‐of‐concept for this approach, highlighting its potential as a promising therapeutic strategy for addressing the unmet needs in TBI treatment. Notably, it overcomes the limitations associated with autologous therapies while offering a targeted intervention that can simultaneously modulate multiple secondary injury pathways.

## 2 Results

### 2.1 Development of EVs‐Loaded BIOGEL for Neural Regeneration

**Figure 2:**
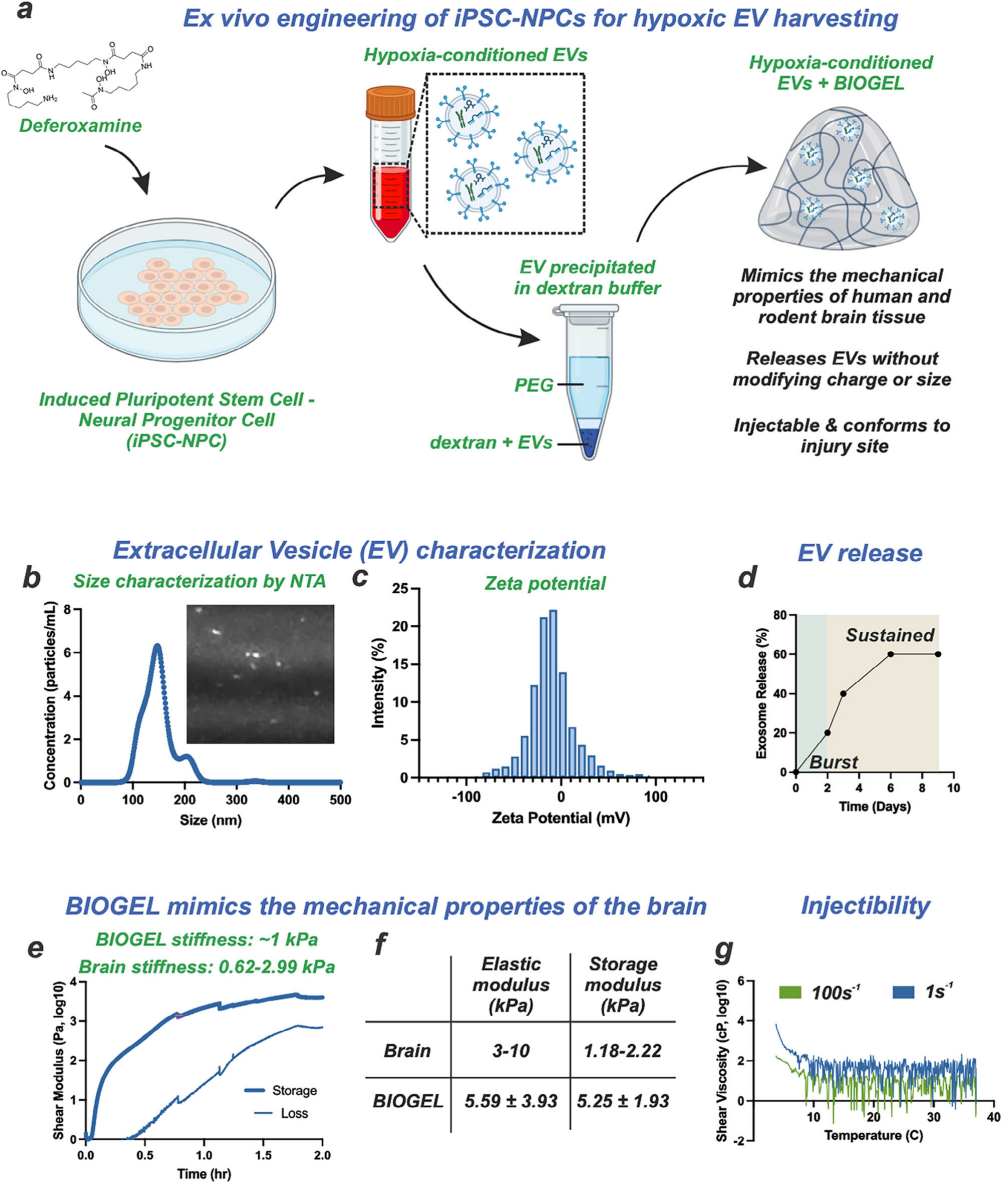
BIOGEL with DFO‐conditioned EVs mimics the mechanical properties of the brain to aid neuronal repair. a) EVs were harvested from the media after 72 h of incubation of DFO with NPCs. b,c) The EVs show the desired size distribution and surface charge. d) A multi‐day release of the EV from the BIOGEL is observed. e) BIOGEL has ideal mechanical properties that aid neuronal migration, proliferation, and differentiation. f) BIOGEL has a similar stiffness and similar elastic and storage modulus to brain tissue. g) Furthermore, a shear viscosity below 3 cP indicates BIOGEL can be administered by injection.

We isolated therapeutic EVs from hiPSC‐NPCs cultured under DFO‐induced hypoxic conditions with maintained cell viability (Figure S1, Supporting Information). Using a standardized isolation protocol (**Figure** **2**a), we characterized the physicochemical properties of the isolated EVs. Nanoparticle tracking analysis revealed a size distribution of 100–250 nm (Figure 2b), while zeta potential measurements showed a negative surface charge, confirming the presence of phospholipid‐enriched membranes (Figure 2c). These physicochemical parameters confirmed the successful isolation of DFO‐conditioned EVs from hiPSC‐NPCs. The isolated EVs maintained stability during extended storage at 4 °C (Figure S2, Supporting Information).

We leveraged our BIOGEL platform to emulate the biomechanical properties of native brain tissue, promoting post‐TBI neuronal regeneration. This was achieved by precisely tuning the concentration of tetrazine and norbornene crosslinking moieties relative to the gelatin precursor during polymer preparation, a methodology investigated in our previous work.^[^ ^17^ ^]^ The EVs proved to be successfully loaded inside the BIOGEL and to undergo sustained release from the gel over six days (Figure 2d). The release is due to a combinatory effect of the EVs diffusing through the hydrogel's porous structure as well as degradation of the hydrogel matrix. BIOGEL's is degraded by Collagenase Type III over a period of ≈50 h.^[^ ^17^ ^]^ For effective CNS regeneration, the scaffold must precisely match brain tissue mechanics to facilitate cellular infiltration while minimizing mechanical stress on the surrounding parenchyma. Rheological characterization demonstrated that BIOGEL's mechanical properties closely approximate those of human brain tissue. The hydrogel exhibits a shear modulus of ≈1 kPa, which falls within the physiological range of brain tissue (0.62–2.99 kPa) (Figure 2e). Similarly, BIOGEL's shear modulus (5.56 kPa) and storage modulus (5.25 kPa) align with reported values for brain tissue (3–10 and 1.18–2.22 kPa, respectively) (Figure 2f).

This biomechanical compatibility underscores BIOGEL's potential as a regenerative platform for TBI treatment, providing an environment conducive to neuronal infiltration and survival. Based on prior evidence showing that scaffolds with an elastic modulus below 10 kPa selectively enhance neuronal survival and growth while inhibiting astrocyte hyper‐proliferation, we hypothesized that BIOGEL—a gelatin‐based hydrogel scaffold—would similarly foster neurogenesis and concurrently attenuate neuroinflammatory immune cell activation within the injury microenvironment.^[^ ^19^ ^]^ To test this hypothesis, we evaluated neuronal response in a co‐culture system of neurons and hypoxic EVs within BIOGEL under neuroinflammatory conditions (Figure S3, Supporting Information). We observed enhanced cell viability, evidenced by increased Hoechst‐positive cells and elevated expression of the neuronal differentiation marker, beta‐III tubulin (TUJ1). The enhanced neuronal differentiation likely stems from the gelatin component of BIOGEL, which provides ECM‐mimetic structural support and activates mechanosensing pathways critical for neuronal development.

In short, our combined in vitro culture experiments and rheological analyses confirm that BIOGEL effectively replicates the mechanical characteristics critical for facilitating neuronal migration, survival, and differentiation. Additionally, the low shear viscosity of BIOGEL, measured at less than 3 × 10^3^ cP, allows for its minimally invasive delivery via injection. This property eliminates the necessity of complex surgical transplantation techniques, offering a streamlined and less invasive therapeutic approach (Figure 2g).

### 2.2 Engineering Hypoxia‐Conditioned Extracellular Vesicles for Neuronal Regeneration

**Figure 3:**
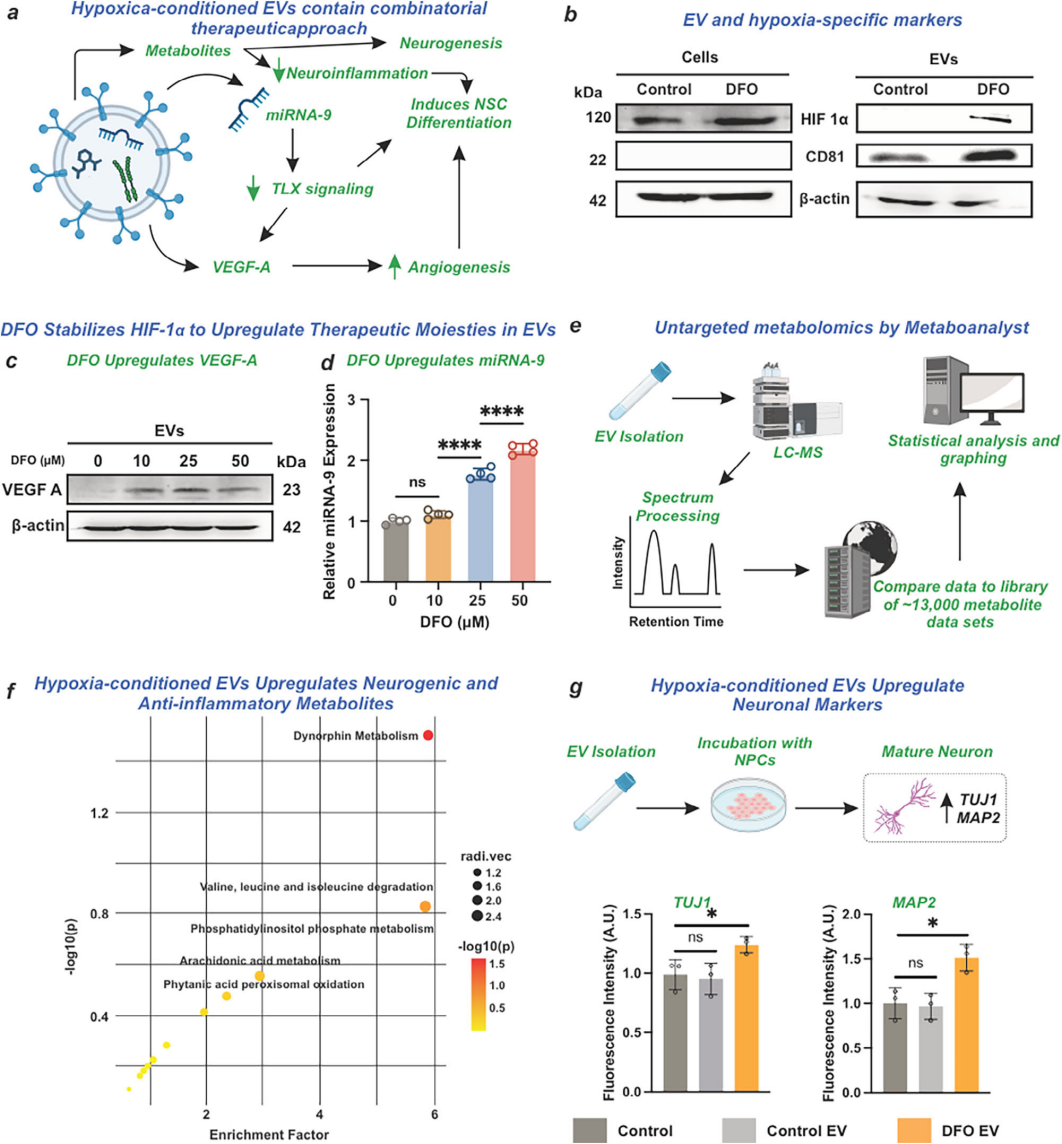
DFO induces hypoxia in NPC to produce EVs with potent wound‐healing factors to aid recovery following TBI. a) EVs endogenously contain proteins, metabolites, and miRNA for injury repair. In our study, DFO upregulated potent wound‐healing factors that enhance angiogenesis and neurogenesis. b) The EVs exhibit specific markers, including CD81 on the EV surface and HIF‐1α for hypoxia. c,d) DFO upregulates VEGF‐A and miRNA‐9. e) An untargeted metabolomic analysis by Metaboanalyst was employed. f) Enrichment analysis using untargeted metabolomics indicates an upregulation of neurogenic and anti‐inflammatory small molecules. g) Healthy NPCs incubated with EVs for 14 days show increased levels of neuronal markers by immunostaining. Ordinary one‐way ANOVA. Unpaired t‐test; ^*^ *p* <  0.05; ^***^ *p* <  0.001; ^****^ *p* <  0.0001, (n = 3).

We systematically engineered EVs under DFO‐induced hypoxic conditions to optimize their neurorestorative potential following TBI (**Figure** **3**a). hiPSC‐NPCs were exposed to DFO for 72 h, followed by EV isolation from conditioned media using a polyethylene glycol (PEG)‐dextran polymer precipitation method. Conditioning with DFO enriches EVs in neuoregenerative factors by upregulating their expression in donor cells, thereby enabling effective delivery while bypassing DFO's limitations of pooor bioavailability, rapid clearance, and off‐target cytotoxicity.^[^ ^20^ ^]^ Analysis of the isolated EVs revealed a size distribution of 50—200 nm, determined by nanoparticle tracking analysis (NTA), and expression of the EV‐specific surface marker CD81, confirmed by Western blot analysis. Our isolation protocol effectively eliminated larger cellular debris and microvesicles. The hypoxic conditioning was validated by the elevated expression of hypoxia‐inducible factor (HIF‐1α) in both parent cells and derived EVs (Figure 3b). We observed a strong concentration‐dependent relationship between DFO treatment and HIF‐1α expression under 30 µm (R^2^ = 0.9856) (Figure S4, Supporting Information), while maintaining cell viability across the EV's therapeutic range (Figure 3c; Figure S5, Supporting Information).

Nuclear translocation of stabilized HIF‐1α triggers the expression of wound healing factors crucial for TBI recovery.^[^ ^21^ ^]^ We focused specifically on two key therapeutic factors: miRNA‐9 and VEGF. HIF‐1α regulates various miRNAs that inhibit neuronal apoptosis and promote synaptic remodeling following TBI.^[^ ^22^ ^]^ Using qPCR analysis, we confirmed the presence of miRNA‐9 in the EVs, with expression levels showing a strong concentration‐dependent response to DFO treatment (R^2^ = 0.9363) (Figure 3d). The microRNA is inversely linked to the TLX transcription factor, which increases angiogenesis and accelerates NSC differentiation.^[^ ^23^ ^]^ The presence of miRNA‐9 is particularly significant due to its dual functions in neural repair. This microRNA promotes neural stem cell differentiation toward neuronal lineages while simultaneously suppressing gliosis, indicating strong neuroregenerative potential.

Beyond its role in miRNA regulation, hypoxia conditioning significantly increased the expression of key angiogenic and neurogenic proteins. Notably, VEGF, a critical factor for promoting neurogenesis in the subventricular zone (SVZ) and subgranular zone (SGZ) of the hippocampus,^[^ ^18^ ^]^ exhibited marked upregulation in hypoxia‐conditioned hNPCs and their derived EVs, as validated by Western blot analysis (Figure 3c). In addition to modulating miRNA expression, DFO treatment increased the levels of proteins involved in angiogenesis and neurogenesis. The simultaneous enrichment of miRNA‐9 and VEGF indicates that hypoxia‐conditioned EVs may effectively promote hippocampal neurogenesis by selectively targeting neural stem cells.

In addition, metabolomic analysis revealed that the EVs contain bioactive metabolites capable of modulating neural stem cell behavior in the hippocampus. We employed liquid chromatography‐mass spectrometry (LC‐MS) to analyze the EV lysate, with metabolite pathway identification performed using the Metabanalyst platform^[^ ^24^ ^]^ (Figure 3e,f; Figure S6, Supporting Information). Among the upregulated pathways, dynorphin metabolism showed the highest enrichment factor (approaching 6). Dynorphin, a 254 amino acid peptide expressed in multiple brain regions, including the hippocampus, acts through kappa opioid receptor (KOR) signaling to suppress neuroinflammation.^[^ ^25^ ^]^ We also observed a significant upregulation of pathways involved in the degradation of valine, leucine, and isoleucine in hypoxia‐conditioned EVs.

These amino acid degradation products serve as critical precursors for the tricarboxylic acid (TCA) cycle, supporting biosynthetic pathways that promote neuronal differentiation by regulating oxidative phosphorylation and sirtuin signaling.^[^ ^26^ ^]^ Additionally, we detected enhanced phosphatidylinositol phosphate metabolism, which activates Protein Kinase C to stimulate neurogenesis in the subgranular zone (SVG) of the hippocampus.^[^ ^27^ ^]^ These metabolomic results demonstrate that DFO conditioning enriches EVs with small‐molecule metabolites capable of promoting neurogenic responses in hippocampal neural stem cells through multiple complementary pathways.

Our characterization data validate the hypoxic conditioning protocol for hiPSC‐NPCs and demonstrate the efficient isolation of EVs from the resulting conditioned media. Characterization of EVs derived from DFO‐conditioned hiPSC‐NPCs revealed a significant enrichment of neurogenic regulatory factors, specifically miRNA‐9 and VEGF. Furthermore, metabolomic analysis identified elevated concentrations of small‐molecule metabolites with documented anti‐inflammatory properties. These molecular signatures suggest that DFO‐conditioned EVs may enhance hippocampal neurogenesis and facilitate neuronal differentiation, though further investigation is needed to establish causative relationships.

### 2.3 Hypoxia‐Conditioned EVs Contain a Multi‐Modal Therapeutic Cargo that Promotes Neuronal Differentiation In Vitro

Comprehensive characterization revealed that isolated EVs contained diverse bioactive molecules potentially beneficial for post‐TBI neuroregeneration. Specifically, these EVs encapsulated proteins, nucleic acids, and metabolites with established anti‐inflammatory and neurogenic properties. To evaluate the neurogenic capacity of DFO‐conditioned EVs, we conducted a 14‐day in vitro study using healthy hiPSC‐NPCs. The DFO‐conditioned EVs were investigated for their intrinsic ability to facilitate neuronal differentiation. Given the extensive characterization of BIOGEL in our prior work, the in vitro experimental design in this study focused on evaluating the novel therapeutic efficacy of the engineered EVs. The immunocytochemical analysis assessed the expression of neuronal differentiation markers across three experimental groups: untreated hiPSC‐NPCs (control), EV‐treated hiPSC‐NPCs (control), and DFO‐conditioned EV‐treated hiPSC‐NPCs (Figure 3g). While treatment with control EVs showed no significant differences compared to untreated controls, hypoxia‐conditioned EVs markedly increased the expression of the early neuronal marker TUJ1 (*p* < 0.0474) and the mature neuronal marker microtubule‐associated protein 2 (MAP2) (*p* < 0.0124). These results strongly suggest that hypoxia‐conditioned EVs actively facilitate neuronal differentiation, as demonstrated by the marked upregulation of both early and mature neuronal markers in vitro. The increased expression of TUJ1 and MAP2 underscores the ability of these EVs to influence key stages of neuronal development, from early differentiation to maturation. We hypothesize that this effect is primarily driven by the bioactive components within the EVs, including miRNA‐9, which regulates neural lineage commitment; VEGF, which promotes angiogenesis and neurogenesis; and a diverse array of small‐molecule metabolites that influence cellular energy dynamics and signaling pathways essential for neuronal growth. Due to the increase in moieties that encourage neurogenesis and neurovascular repair, coupled with the fact that control EVs did not significantly increase vital neurogenic markers, we hypothesize that the DFO conditioning of the EVs is paramount for effective injury repair.

In parallel, the observed lack of significant differences between untreated controls and cells treated with control EVs further emphasizes the unique impact of hypoxia‐conditioned EVs. This highlights the critical role of hypoxia‐driven HIF‐1α stabilization in enriching EVs with potent neurotrophic factors. HIF‐1α acts as a master regulator of the cellular hypoxic response, inducing the production of key growth factors and signaling molecules that enhance the therapeutic efficacy of the EV cargo. Together, these findings indicate that the hypoxia‐conditioned state not only enhances the regenerative potential of EVs but also underscores the importance of environmental conditioning in optimizing EV‐based therapies for neural repair and regeneration.

### 2.4 Reduction in Lesion Volume Following TBI with EV‐Loaded BIOGEL Treatment

**Figure 4:**
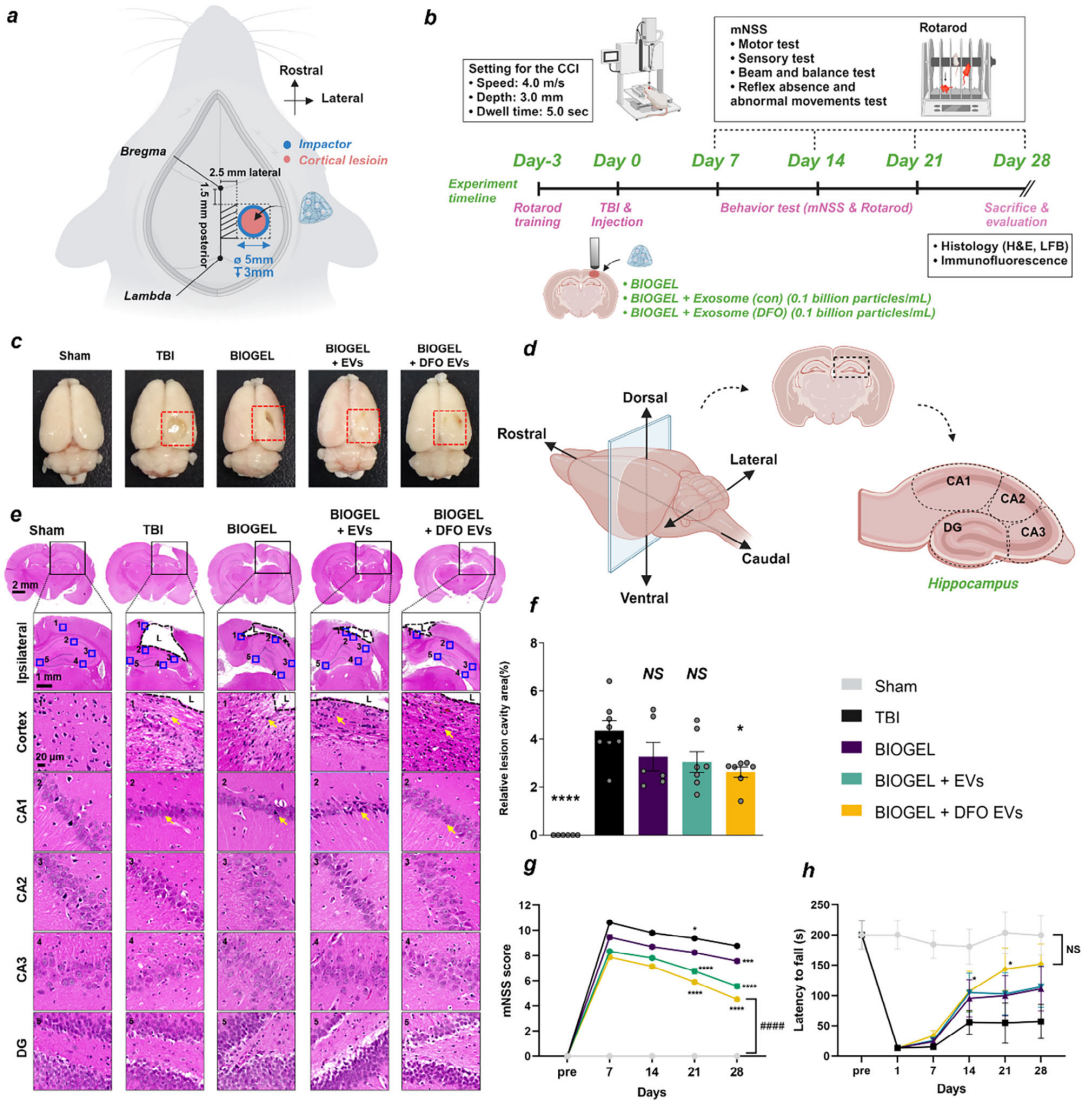
BIOGEL‐delivered DFO‐conditioned EVs promote functional recovery following TBI. a) Schematic illustration of craniotomy site and therapeutic delivery locations for BIOGEL alone, control EV‐loaded BIOGEL (BIOGEL +EVs), and DFO‐conditioned EV‐loaded BIOGEL (BIOGEL + DFO EVs) following controlled cortical impact (CCI). b) An experimental timeline from the CCI procedure through behavioral assessments will be used to study the endpoint. Created with BioRender.com. c) Representative gross anatomical images of rat brains across experimental groups. Red dashed boxes indicate regions of cortical injury. d) Schematic representation of hippocampal anatomical subregions. e) Representative H&E‐stained sections of the cortex and hippocampal subregions (CA1, CA2, CA3, dentate gyrus) from sham, untreated TBI, and treatment groups. Yellow arrows indicate altered cellular morphology. Scale bars: 2 mm (whole brain), 1 mm (ipsilateral regions), 20 µm (hippocampal regions). f) Quantification of cortical lesion volume normalized to total brain volume. n = 8 (TBI), n = 7 (EV‐loaded hydrogel groups), n = 6 (sham and hydrogel alone). ^*^ *p* < 0.05, ^****^ *p* < 0.0001, NS: not significant versus TBI group; one‐way ANOVA with Tukey's post hoc test. g) Modified Neurological Severity Score (mNSS) was assessed weekly over 28 days post‐injury. n = 8 (sham and TBI), n = 10 (treatment groups). ^*^ *p* < 0.05, ^***^ *p* < 0.001, ^****^ *p* < 0.0001 versus TBI group; two‐way ANOVA with Bonferroni's post hoc test. In mNSS assessment, asterisks (^*^) indicate statistical differences versus the TBI group, while “#” indicates statistical differences versus the sham group (#### *p* < 0.0001). h) Rotarod performance was evaluated weekly over 28 days post‐injury. n = 8 (sham and TBI), n = 10 (treatment groups). ^*^ *p* < 0.05 versus TBI group; two‐way ANOVA. Data presented as mean ± SEM.

To assess the therapeutic potential of our biomaterial platform, we utilized a Controlled Cortical Impact (CCI) model, a well‐established method replicating the key pathological features of moderate‐to‐severe TBI. This model effectively mimics critical aspects of TBI, including cortical contusions, subarachnoid hemorrhages, and associated neurological impairments, providing a robust framework for evaluating treatment efficacy (**Figure** **4**a). Female Sprague‐Dawley rats were randomized into five experimental groups: sham operation, untreated TBI, BIOGEL‐treated TBI, control EV‐loaded BIOGEL‐treated TBI, and DFO‐conditioned EV‐loaded BIOGEL‐treated TBI. A concentration of 1.0 × 10^8^ particles per EV was decided to observe the relevant therapeutic properties of the EV without inducing toxicity. Prior to CCI surgery, baseline motor function was established using rotarod assessment. Post‐injury motor function was evaluated weekly for 28 days. Following terminal endpoints, therapeutic outcomes were assessed through comprehensive histological and immunofluorescence analyses (Figure 4b). These groups correlated with our in vitro test in Figure 3g to validate the EV's effectiveness in promoting neuronal repair.

Initial assessment of injury severity focused on quantifying lesion volume. All TBI groups exhibited significant cortical damage compared to sham controls (Figure 4c). Hippocampal anatomy and subregions are illustrated in (Figure 4d). Hematoxylin and eosin (H&E) staining enabled quantification of the lesion‐to‐ipsilateral cortex ratio and evaluation of hippocampal neuronal morphology (Figure 4e). Quantitative analysis revealed that DFO‐conditioned EV‐loaded BIOGEL treatment significantly reduced lesion volume (2.62% ± 0.57 of total brain area) compared to untreated TBI controls (4.35% ± 1.2) (Figure 4f). We assessed behavioral outcomes over 28 days using the modified Neurological Severity Score (mNSS) and Rotarod testing to evaluate whether reduced tissue damage corresponded with functional improvement. DFO‐conditioned EV‐loaded BIOGEL treatment significantly improved performance in both measures. The treatment group demonstrated lower mNSS scores (4.53 ± 0.36) compared to untreated TBI controls (8.77 ± 0.35), indicating enhanced neurological function (Figure 4g). Similarly, Rotarod performance in the treatment group (152.125 ± 104.997 s) approached sham control levels (199.708 ± 92.26 s) (Figure 4h). The superior outcomes achieved with hypoxia‐conditioned EV‐loaded BIOGEL, compared to BIOGEL alone or control EV‐loaded BIOGEL, indicate a synergistic effect arising from the combined treatment approach. These findings align with previous studies, which show that while the hydrogel component provides structural support conducive to neuronal survival and proliferation, the hypoxia‐conditioned EVs deliver specific neurogenic factors that actively drive neuronal differentiation. The minimal improvements seen in the BIOGEL‐only and control EV‐loaded BIOGEL groups highlight the essential function of hypoxia‐induced HIF‐1α stabilization in promoting EVs with regenerative factors crucial for therapeutic enhancement efficacy. Similar trends were observed when the BIOGEL was administered on day 7 following injury (Figure S7, Supporting Information).

These findings prove that the delivery of hypoxia‐conditioned EVs via BIOGEL effectively promotes tissue regeneration following TBI. The observed preservation of neural tissue suggests a promising potential for restoring disrupted neural circuits. Recognizing the pivotal role of neuroinflammation in the progression of secondary injury, we subsequently investigated whether this therapeutic platform could modulate the immune response, creating a more favorable microenvironment for neural repair and regeneration.

### 2.5 Hypoxia‐Conditioned EV‐Loaded BIOGEL Modulates the Inflammatory Response and Enhances ECM Remodeling

**Figure 5:**
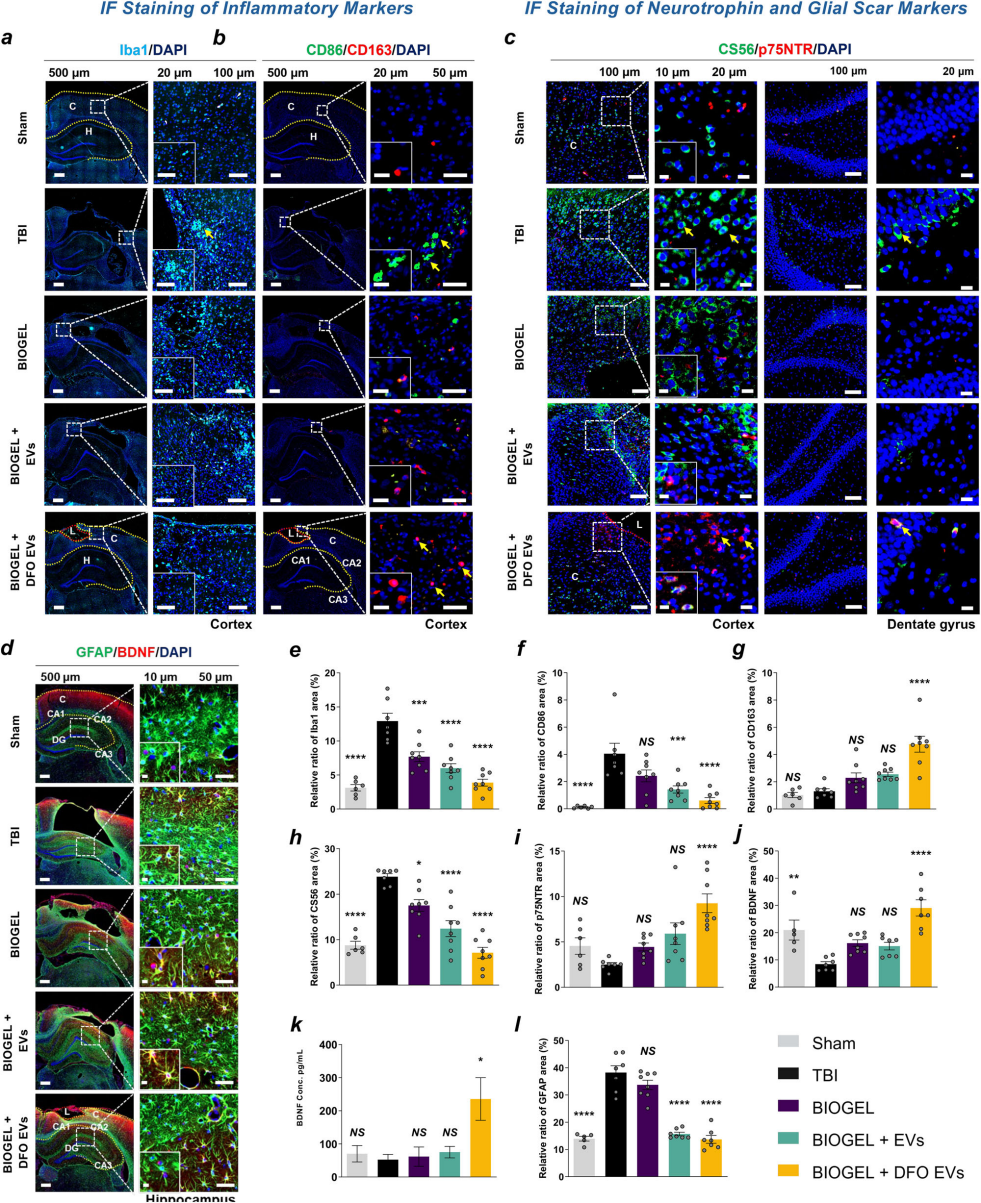
DFO‐conditioned EV‐loaded BIOGEL modulates neuroinflammation and promotes ECM remodeling. a,b) Representative immunofluorescence images of cortical tissue showing Iba1 (cyan), CD86 (green), CD163 (red), and nuclear counterstain DAPI (blue). c–h) Quantification of immunofluorescence intensity for inflammatory markers Iba1, CD86, CD163, ECM components CS56, neurotrophic signaling p75NTR, and BDNF. n = 8 (TBI), n = 7 (EV‐loaded hydrogel groups), n = 6 (sham and hydrogel alone). i) Representative immunofluorescence images of the lesion site showing CS56 (green), p75NTR (red), and DAPI (blue). Yellow arrowheads indicate regions of positive immunoreactivity. j) Representative immunofluorescence images of the hippocampus showing GFAP (green), BDNF (red), and DAPI (blue). C: Cortex, L: Lesion site, H: Hippocampus. k) BDNF protein quantification by ELISA across experimental groups. l) Quantification of immunofluorescence intensity for reactive gliosis (GFAP) across experimental groups. n = 8 (TBI), n = 7 (EV‐loaded hydrogel groups), n = 6 (sham and hydrogel alone). ^*^ *p* < 0.05, ^**^ *p* < 0.01, ^***^ *p* < 0.001, ^****^ *p* < 0.0001, NS: not significant versus TBI group; one‐way ANOVA with Tukey's post hoc test. Data presented as mean ± SEM.

The brain's extracellular matrix (ECM) provides an essential scaffold that is critical for maintaining tissue homeostasis. Beyond its structural role, the ECM actively orchestrates intercellular communication and delivers biomechanical cues that regulate cellular behavior. To systematically investigate injury‐induced ECM reorganization and its interplay with neuroinflammation, we analyzed immune cells for cytokine levels following treatment with BIOGEL scaffold loaded with DFO‐derived EVs (BIOGEL + DFO EVs) in a rodent TBI model. In untreated TBI, activated microglia release neurotoxic factors that promote ECM degradation and impair tissue repair. This inflammatory response is typically accompanied by the recruitment of fibroblasts and reactive astrocytes, leading to excessive ECM deposition and glial scar formation. DFO‐conditioned EV‐loaded BIOGEL treatment significantly reduced inflammatory markers at the injury site. Specifically, expression of the microglial activation marker Iba1 (**Figure** **5**a–c) decreased substantially in the treatment group (3.87% ± 1.34) compared to untreated TBI controls (12.921% ± 2.813), indicating attenuated microglial activation post‐injury. Macrophage polarization exhibited a similar trend, with distinct shifts observed following injury. Infiltrating macrophages generally display a mix of pro‐inflammatory (M1) and anti‐inflammatory (M2) phenotypes.

However, under pathological conditions associated with trauma, these cells predominantly skew toward a pro‐inflammatory M1 state, exacerbating tissue damage and impeding recovery.^[^ ^28^ ^]^

DFO‐conditioned EV (BIOGEL + DFO EVs) treatment modulated this phenotypic distribution, as demonstrated by decreased expression of the M1 marker CD86 and increased expression of the M2 marker CD163 (Figure 5b). Quantitative analysis revealed significantly reduced CD86 expression in the DFO‐conditioned EV‐loaded BIOGEL group (0.611% ± 0.562) compared to untreated TBI controls (4.046% ± 1.885). Conversely, CD163 expression increased in the treatment group (4.758% ± 1.539) relative to TBI controls (1.303% ± 0.457) (Figure 5d,e). Remarkably, subacute administration of DFO‐conditioned EV‐loaded BIOGEL modulated macrophage phenotype, as evidenced by altered CD86/CD163 expression profiles, despite reduced baseline neuroinflammation at this time‐point (Figure S8a–e, Supporting Information). This shift in macrophage phenotype toward a more anti‐inflammatory state indicates the establishment of a microenvironment that supports ECM homeostasis and facilitates tissue repair, creating conditions conducive to regenerative processes.

The hippocampus is particularly vulnerable to damage from TBI. Following such an injury, the resulting glial and fibrotic responses are largely regulated by chondroitin sulfate proteoglycans (CSPGs). These molecules, which are produced by neural cells and function as both ECM components and transmembrane proteins, are therefore critical players in the post‐injury environment. Immunofluorescence analysis revealed region‐specific CSPG expression (CS56), with a minimal presence in the dentate gyrus but marked expression at the cortical lesion site during both acute and subacute phases (Figure 5i; Figure S8f–k, Supporting Information). DFO‐conditioned EV‐loaded BIOGEL treatment significantly reduced CS56 expression (7.114% ± 3.186) compared to untreated TBI controls (23.849% ± 1.602) (Figure 5f).

To evaluate neurotrophic signaling during ECM remodeling, we analyzed the expression of BDNF and its receptor p75NTR. Treatment with DFO‐conditioned EV‐loaded BIOGEL significantly upregulated cortical p75NTR expression (9.251% ± 2.731) (Figure 5g). BDNF immunoreactivity was most pronounced in the hippocampus of the treatment group (Figure 5k–h). ELISA quantification revealed a significant increase in BDNF levels in the hypoxia‐conditioned EV‐loaded BIOGEL treatment group (235.640 ± 128.702 pg mL^−1^) compared to the TBI control group (52.322 ± 31.824 pg mL^−1^) (Figure 5i). This elevation in BDNF suggests enhanced neurotrophic support, which is critical for promoting ECM regeneration and facilitating neural repair. Quantitative analysis of glial fibrillary acidic protein (GFAP), a hallmark of reactive astrogliosis and glial scarring, revealed widespread bilateral expression in both cortical and hippocampal regions post‐TBI, particularly proximal to the lesion site. Treatment with DFO‐conditioned EV‐loaded BIOGEL resulted in a significant reduction of GFAP expression (13.689% ± 3.186) compared to untreated controls (38.222% ± 6.173), showing diminished astrocyte reactivity and suppressed glial scar formation (Figure 5i).

Concurrently, supplementary testing revealed brain ECM remodeling and recovery, as indicated by a reduction in both glial and fibrotic scarring (Figure S8k–m, Supporting Information). These results show that hypoxia‐conditioned EV‐loaded BIOGEL treatment effectively modulates multiple aspects of the post‐injury response. This includes alterations in immune cell phenotypes, reductions in inflammatory marker expression, remodeling of the ECM, and decreased astrocyte reactivity. Collectively, these changes contribute to the creation of a microenvironment that is more favorable for neural repair. Previous research has shown that such immunomodulation can lower levels of pro‐inflammatory mediators and neurotoxic factors, such as IL‐1β, nitric oxide, and hydrogen peroxide, which are known to exacerbate secondary injury. Building on these insights, we next investigated whether the enhanced tissue environment established by this treatment supports improved neuronal regeneration.

### 2.6 Hypoxia‐Conditioned EV‐Loaded BIOGEL Enhances Neural Stem Cell Proliferation and Promotes Neurogenesis

**Figure 6:**
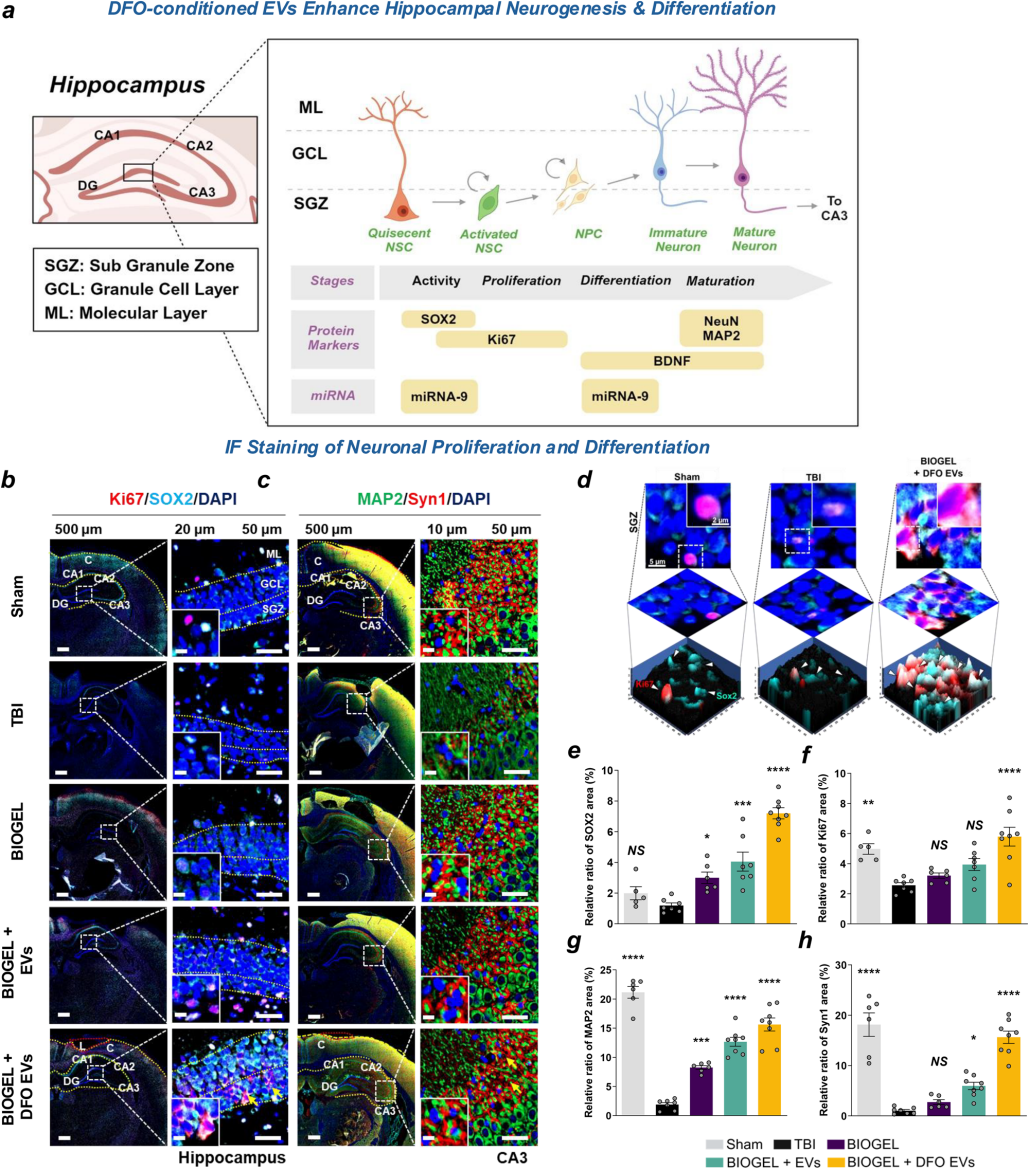
DFO‐conditioned EV‐loaded BIOGEL promotes hippocampal neurogenesis and neuronal differentiation. a) Schematic illustration of hippocampal anatomy, highlighting subregions CA1, CA2, CA3, and dentate gyrus, with emphasis on the subgranular zone (SGZ) neurogenic niche. Sequential stages of neurogenesis are shown, depicting miRNA‐9‐mediated regulation of neural progenitor proliferation and differentiation. b) Representative immunofluorescence images showing Ki67 (red) and SOX2 (cyan) expression with DAPI nuclear counterstain (blue) in hippocampal sections. c) Representative immunofluorescence images showing MAP2 (green) and Synapsin1 (red) expression with DAPI nuclear counterstain (blue). C: Cortex, L: Lesion site, H: Hippocampus. Yellow arrowheads indicate regions of positive immunoreactivity. d) High‐magnification 3D reconstructions of SOX2/Ki67‐positive cells in the SGZ across experimental groups. e–h) Quantification of immunofluorescence intensity for Ki67, SOX2, MAP2, and Synapsin1. n = 8 (TBI), n = 7 (EV‐loaded hydrogel groups), n = 6 (sham and hydrogel alone). ^*^ *p* < 0.05, ^**^ *p* < 0.01, ^***^ *p* < 0.001, ^****^ *p* < 0.0001, NS: not significant versus TBI group; one‐way ANOVA with Tukey's post hoc test. Data presented as mean ± SEM.

To assess neurogenic effects, we focused on the SGZ of the dentate gyrus, a key neurogenic niche in the rodent brain that generates new granule neurons essential for hippocampal‐dependent cognitive functions (**Figure** **6**a). SGZ neurogenesis is crucial for maintaining hippocampal functionality, which is achieved by continually supplying new neurons to the dentate gyrus. For this analysis, we examined two critical markers: SOX2, a transcription factor that identifies multipotent neural stem and progenitor cells and is essential for regulating oligodendrocyte progenitor cell (OPC) proliferation and survival, and Ki67, a nuclear protein that marks cells in active phases of the cell cycle, indicating cell proliferation (Figure 6b). Following TBI, SOX2 expression decreased substantially in the hippocampus, particularly within the SGZ. DFO‐conditioned EV‐loaded BIOGEL treatment significantly increased SOX2 expression (7.197% ± 1.025) compared to untreated TBI controls (1.19% ± 0.409) (Figure 6e). Ki67 expression showed a similar pattern, with elevated levels in the treatment group (5.796% ± 1.65) (Figure 6f). High‐magnification quantitative analysis of SOX2 and Ki67 expressions in the SGZ demonstrated significantly enhanced activation of neural stem and progenitor cells in the hypoxia‐conditioned EV‐loaded BIOGEL treatment group (Figure 6d).

Treatment with DFO‐conditioned EV‐loaded BIOGEL significantly upregulated the neurogenesis markers Ki67 and SOX2, further supporting these findings (Figure S9a–d, Supporting Information). The hypoxia‐induced upregulation of miRNA‐9 appears to perform dual functions: maintaining neural stem cell quiescence to preserve the progenitor pool and coordinating with other miRNAs to facilitate the differentiation of neural stem cells into intermediate progenitor cells and neuroblasts,^[^ ^29^ ^]^ likely stems from the synergistic interaction between the supportive microenvironment provided by BIOGEL and the regulatory factors encapsulated within the EVs, which collectively enhance neurogenic processes.

Neuronal maturation and synaptic integration in the dentate gyrus and CA3 regions were evaluated using two established markers: MAP2 for dendritic cytoskeletal organization and Synapsin1 (Syn1) for presynaptic vesicle dynamics. TBI significantly reduced both markers in the CA3 region (Figure 6c). Quantitative analysis of defined regions of interest (ROIs) demonstrated that DFO‐conditioned EV‐loaded BIOGEL treatment significantly increased MAP2 expression (15.631% ± 2.95) compared to untreated TBI controls (1.896% ± 0.814) (Figure 6g). Similarly, Syn1 expression was markedly elevated in the treatment group (Figure 6h).

Our findings highlight enhanced neural regeneration driven by the consistent promotion of neurogenesis. The observed upregulation of key markers, including SOX2 and Ki67, signifies increased neural stem cell proliferation and activation, while elevated levels of MAP2 and Synapsin1 reflect advanced neuronal differentiation and synaptic integration. Because remyelination is critical for functional recovery after neural injury, and given that the platform's biomaterial properties were previously shown to preferentially promote neurogenesis over gliosis, we investigated whether oligodendrocyte‐mediated remyelination contributes to the observed therapeutic benefits.

Hypoxia‐conditioned EV‐loaded BIOGEL treatment significantly promoted oligodendrocyte‐mediated remyelination in the brain TBI, indicating its potential to restore neural connectivity and functionality. Luxol Fast Blue staining assessed myelin integrity (Figure S9a, Supporting Information), with the treatment group showing significantly increased remyelination compared to untreated TBI controls (*p* < 0.0001) (Figure S9b, Supporting Information). We examined multiple markers: SOX10, expressed during OPCs development through maturation; myelin basic protein (MBP) in oligodendrocyte myelin sheaths; neuron‐specific TUJ1 for early neuronal development; and neuronal nuclear antigen (NeuN) for mature neurons (Figure S9d,e, Supporting Information). In the peri‐lesional cortex, the untreated TBI controls exhibited minimal presence of oligodendrocytes.

In contrast, the hypoxia‐conditioned EV‐loaded BIOGEL treatment group showed a substantial increase in oligodendrocyte populations, corresponding with elevated neural stem cell activity observed in the dentate gyrus. The treatment group exhibited both SOX10 and TUJ1 expressions, indicating active OPC development. Quantitative analysis revealed significantly higher SOX10 expression in the SGZ with DFO‐conditioned EV‐loaded hydrogel treatment (17.692% ± 3.232) compared to untreated TBI controls (7.640% ± 1.947) (Figure S9c,d, Supporting Information). Similarly, TUJ1 expression increased significantly with treatment (14.844% ± 4.210 vs 4.475% ± 2.430) (Figure S9d,f, Supporting Information). NeuN expression showed a marked elevation in the treatment group (17.191% ± 3.899) compared to TBI controls (6.292% ± 1.119) (Figure S9e,g, Supporting Information). MBP expression demonstrated the most pronounced difference between treatment and TBI groups (19.941% ± 2.055 vs 2.483% ± 1.270) (Figure 9h). Similar results were observed in the delayed treatment group (Figure S10, Supporting Information).

These results show that DFO‐conditioned EV‐loaded BIOGEL treatment enhances both remyelination and neuronal maturation following TBI. Consistently, subacute administration of the therapeutic platform maintained comparable expression patterns of the neuronal marker TUJ1, the oligodendrocyte transcription factor Sox10, and the myelin protein MBP (Figure S11, Supporting Information).

The enhanced neural regeneration and functional recovery observed in this study highlight the therapeutic potential of hypoxia‐conditioned EV‐loaded BIOGEL for treating TBI. The elevated expression of TUJ1 and SOX10 reflects improved neuronal differentiation, while the increased oligodendrocyte populations and neuron‐oligodendrocyte co‐localization point to enhanced myelination. Taken together, these findings on synaptic integration, combined with the previously demonstrated reduction in neuroinflammation (Section 2.5), strongly suggest that our BIOGEL‐EV platform facilitates the repair of neural circuits. This dual‐action approach, which addresses both inflammatory and regenerative pathways, represents a potentially novel strategy for overcoming the multifaceted challenges of TBI recovery.

### 2.7 Hypoxia‐Conditioned EV‐Loaded BIOGEL Promotes Angiogenesis and Restores Blood‐Brain Barrier Integrity

We next assessed the platform's ability to improve ischemia and restore the integrity of the BBB (Figure S12a, Supporting Information). Vascular dysfunction and BBB disruption following TBI are critical pathological events that can significantly impair neuronal function and hinder tissue regeneration. We analyzed cerebral microvascular endothelial cells using two markers: CD31 (platelet endothelial cell adhesion molecule‐1) for vascular endothelium and occludin for tight junctions. CD31 expression was significantly elevated in the peri‐lesional cortex following DFO‐conditioned EV‐loaded BIOGEL treatment (15.640% ± 2.653) compared to untreated TBI controls (3.806% ± 1.060), indicating enhanced vascularization (Figure S12b, Supporting Information).

Similarly, occludin expression increased significantly with DFO‐conditioned EV‐loaded BIOGEL treatment (9.241% ± 2.057) compared to TBI controls (3.484% ± 1.111), suggesting improved BBB integrity (Figure S12c, Supporting Information). Improved vascularization and blood‐brain barrier (BBB) restoration facilitate the re‐establishment of molecular and ionic gradients, which are essential for maintaining neural function and promoting tissue recovery. The observed beneficial effects are likely a result of the VEGF delivered by EVs, a critical factor in endothelial cell proliferation, migration, and survival, as well as in regulating vascular permeability and maturation via receptor‐mediated signaling. We hypothesize that the observed tissue repair is a consequence of the combined effects of EV‐delivered VEGF and the hydrogel's restorative properties, which work together to support and restore the structural integrity of the ECM.

Treatment with hypoxia‐conditioned EV‐loaded BIOGEL demonstrated a range of therapeutic benefits following TBI, including enhanced angiogenesis, restoration of blood‐brain barrier (BBB) integrity, reduction of neuroinflammation, and improved functional recovery. Vascular regeneration and BBB restoration are essential elements of post‐injury neural repair. Immunofluorescence analysis of the endothelial marker CD31 and the tight junction protein Occludin revealed that hypoxia‐conditioned EV‐loaded hydrogel treatment resulted in the most significant improvements in both vascularization and BBB integrity, underscoring its potential for effective neural repair.

## 3 Conclusion

In this study, we successfully demonstrate that an engineered gelatin‐based hydrogel scaffold (BIOGEL) enables the sustained delivery of extracellular vesicles from human‐induced pluripotent stem cell‐derived neural progenitor cells (hiPSC‐NPC EVs). In a rodent model of TBI, this platform synergistically enhanced neurogenesis and modulated key neuroinflammatory pathways, representing a significant translational advance for central nervous system (CNS) repair. By conditioning the parent cells under hypoxic conditions to mimic the post‐injury microenvironment, the EVs were enriched with critical neurogenic and angiogenic factors, including miRNA‐9 and VEGF. Encapsulation of these EVs within BIOGEL enabled controlled and sustained release, promoting their therapeutic efficacy while maintaining the structural and biochemical properties of native brain tissue.

We initially investigated the therapeutic efficacy of DFO‐conditioned EV‐loaded BIOGEL during the acute phase of TBI via immediate post‐injury administration in vivo. This intervention strategy targeted early secondary injury cascades, particularly neuroinflammation, while promoting neuronal maturation and differentiation. Our research results strongly supported and confirmed the findings of previous studies, which clearly demonstrated BIOGEL's unique dual functionality, acting as both an effective anti‐inflammatory agent and a highly versatile therapeutic delivery system. To elucidate the temporal therapeutic window, we extended our investigation to the subacute phase through DPI‐7 administration, capitalizing on the partially restored BBB integrity and diminished inflammatory environment for enhanced EV retention and efficacy. Remarkably, both acute and subacute interventions demonstrated comparable efficacy in attenuating glial scarring and preserving cytoarchitecture in the trauma‐susceptible cortical and hippocampal regions. Significant reductions in lesion volume and improvements in structural preservation were observed in those areas particularly susceptible to primary and secondary injury mechanisms. This study also shows that the novel BIOGEL‐EV therapeutic platform effectively addresses two critical challenges in TBI treatment: mitigating secondary injury mechanisms, such as inflammation and glial scar formation, and providing structural support during the repair process. This combined approach offers substantial promise for improving functional outcomes and enhancing recovery following TBI by establishing a pro‐regenerative microenvironment.

Secondary injury following TBI is marked by glial scar formation, which establishes physical and biochemical barriers that hinder axonal regeneration. The results of our study also clearly indicate that the use of BIOGEL treatment, which incorporates hypoxia‐conditioned extracellular vesicles, leads to a substantial reduction in lesion volume and a notable decrease in the buildup of CSPGs, or chondroitin sulfate proteoglycans. While CSPGs are known to contribute to scarring and inhibit neurogenesis, the hypoxia‐conditioned EVs modulated their deposition within the lesion core, fostering a more permissive environment for neural repair and regeneration.

The modified EVs markedly diminished reactive astrogliosis, indicating a successful reprogramming of the injury microenvironment toward a regenerative state. This was further supported by neurotrophic signaling mediated through p75NTR‐BDNF interactions, which enhanced endogenous recovery mechanisms, promoting neuronal survival and synaptic plasticity. While p75NTR has a relatively low affinity for BDNF, the elevated neurotrophin levels effectively activated TRK‐mediated signaling pathways, which are essential for functional restoration in the dentate gyrus and CA3 regions of the hippocampus.

The treatment effectively promoted neurogenesis and remyelination, as evidenced by the upregulation of key molecular markers. Increased expression of SOX2 and Ki67 in the subgranular zone indicated enhanced proliferation of neural stem and progenitor cells, while elevated levels of myelin basic protein (MBP) reflected significant remyelination. These findings highlight a coordinated cellular repair process addressing both neuronal loss and myelin restoration, which are crucial for functional recovery. Moreover, the upregulation of dendritic and synaptic markers, MAP2 and Synapsin1, demonstrated improved neural connectivity, correlating with enhanced motor function. This treatment approach also significantly enhanced vascular function and BBB integrity. Increased angiogenesis and restoration of the BBB were critical in re‐establishing a supportive neural microenvironment, reducing inflammation, and facilitating the targeted delivery of reparative factors to the injury site. Furthermore, the observed shift in microglial phenotypes from pro‐inflammatory (M1) to anti‐inflammatory (M2) states underscores the immunomodulatory effects of the treatment, contributing to the creation of a microenvironment conducive to tissue repair and regeneration.

The significant improvements observed in behavioral outcomes offer robust quantitative validation of the treatment's efficacy, demonstrating the successful translation of molecular and cellular repair into functional recovery. These excellent therapeutic results highlight the potential of hypoxia‐conditioned EV‐loaded BIOGEL to address both the structural and cellular consequences of TBI while paving the way for improved clinical outcomes. Our demonstration may provide new insights into post‐TBI recovery mechanisms and establish a strong foundation for future therapeutic development. The synergistic interaction between BIOGEL and hypoxia‐conditioned EVs represents an innovative platform for further exploration, including optimization of delivery systems, more detailed mechanistic studies, and evaluation for clinical translation. These findings mark a significant advance in developing targeted, multifaceted interventions for TBI, with broader implications for advancing regenerative medicine strategies in treating neurotrauma.

In conclusion, this study highlights the therapeutic potential of hypoxia‐conditioned EV‐loaded BIOGEL as a multifaceted approach to addressing TBI. Our therapeutic strategy utilized EVs isolated from hypoxia‐conditioned hiPSC‐NPCs. These EVs were selectively enriched with miRNA‐9, a neurogenic regulator, and vascular endothelial growth factor (VEGF), an angiogenic factor—two key mediators of neuronal differentiation and blood‐brain barrier (BBB) restoration. The BIOGEL platform facilitated the sustained release of EVs while simultaneously inhibiting chondroitin sulfate proteoglycan synthesis, a key barrier to neural regeneration. In a rodent model of TBI, treatment with hypoxia‐conditioned EV‐loaded BIOGEL significantly enhanced hippocampal neurogenesis and preserved oligodendrocytes, while also reducing neuroinflammation. The intervention led to decreased cortical lesion volumes, restored BBB integrity, and improved behavioral outcomes, proving its ability to promote structural repair and functional recovery. Together, these findings demonstrate that the BIOGEL scaffold, delivering hypoxia‐primed hiPSC‐NPC EVs, can form a modular therapeutic platform capable of simultaneously targeting neuroinflammation, vascular dysfunction, and neural circuit repair in TBI. This combinatorial approach could eventually address the multifaceted pathophysiology of TBI, offering a translatable strategy to advance regenerative medicine paradigms and improve functional outcomes in neurotrauma care.

## 4 Experimental Section

### Synthesis of BIOGEL Precursors and BIOGEL Formation

The BIOGEL precursors, GelTz and GelNb, were synthesized following the protocols described in the previous work.^[^ ^17^ ^]^

### Extracellular Vesicles (EVs) Isolation and Characterization

Deferoxamine mesylate (DFO) was dissolved in Dulbecco's phosphate‐buffered saline (DPBS, 10 mm), sterilized through a 0.22 µm filter, and added to iPSC‐derived neural progenitor cell media. Cells were cultured in control or DFO‐supplemented media for 72 h before media collection.

EVs were isolated through differential centrifugation, followed by polymer precipitation. Conditioned media underwent sequential centrifugation at 4 °C to remove cellular debris: 500 × g (10 min), 2000 × g (10 min), and 10000 × g (30 min). The supernatant was incubated overnight at 4 °C with polyethylene glycol (PEG, MW 20 000) and dextran (MW 450 000—650 000) at final concentrations of 10% w/v each in DPBS. Following incubation, the mixture was centrifuged at 1000 × g (10 min) to separate the PEG phase, and the remaining solution was centrifuged at 13 000 × g (10 min) to pellet EVs.

The EV pellet was resuspended in DPBS, sterilized through a 0.22 µm filter, and analyzed by NTA for particle concentration determination.

### Biophysical Characterization of EVs—Sample Preparation

EV suspensions were diluted in DPBS to 10^6^–10^7^ particles mL^−1^ for all analyses.

### Biophysical Characterization of EVs—Dynamic Light Scattering and Zeta Potential

Diluted EV samples were analyzed in spectrophotometer cuvettes and folded capillary cells using a ZetaSizer Nano NS (Malvern Panalytical, UK) for size distribution and surface charge measurements.

### Biophysical Characterization of EVs—Nanoparticle Tracking Analysis

Particle size distribution and concentration were determined using a NanoSight NS300 (Malvern Panalytical, UK) equipped with a flow cell system.

### Preparation of EV‐Loaded BIOGEL—Precursor Solution Preparation

GelTz and GelNb were reconstituted in DPBS at 5% and 10% w/v, respectively. The GelNb solution was combined with EV suspension to achieve the target EV concentration at a final GelNb concentration of 5% w/v.

### Preparation of EV‐Loaded BIOGEL—BIOGEL Formation

Equal volumes of GelTz and diluted GelNb/EV solutions (25 µL each) were mixed and immediately transferred to cylindrical molds (5 mm diameter × 8 mm height) created in Parafilm. Hydrogels were allowed to cross‐link at room temperature for 15 min.

### EVs Release Kinetics—Fluorescent Labeling of EVs

Isolated EVs were suspended in 400 µL DPBS. Atto 633‐NHS ester (10 mm, 1 µL) was added to EV suspension (20 µL) and incubated overnight at 4 °C. Labeled EVs were purified using 7 kDa MWCO Zebra Spin Desalting Columns (Thermo Scientific, cat. 89882).

### EVs Release Kinetics—Release Profile Analysis

To assess release kinetics, labeled EVs were encapsulated in hydrogels and suspended in 500 µL DPBS. Aliquots (50 µL) were collected at predetermined time points. EV release was quantified by measuring fluorescence intensity using a Tecan Infinite M200 Pro plate reader (excitation: 630 nm, emission: 662 nm). Fluorescence values were normalized to initial measurements.

### Rheological Characterization

Rheological characterization was performed using a Kinexus Ultra rotational rheometer (Malvern Panalytical, UK) equipped with parallel stainless‐steel plates (20 mm diameter, 0.6 mm gap), with 200 µL sample volumes loaded for each measurement. The crosslinking kinetics of the hydrogels were monitored using oscillatory rheometry. Freshly mixed GelTz and GelNb solutions (5% w/v in PBS) were analyzed at 37 °C under a frequency of 1 Hz and a shear strain of 1% for two hours. The mechanical properties of the cross‐linked hydrogels were evaluated through frequency sweeps (0.01–10 Hz at 37 °C, 1% strain) and amplitude sweeps (0.1–10% shear strain at 37 °C, 1 Hz). The injectability of the precursor solutions was assessed using viscometry during temperature sweeps from 37 to 4 °C (cooling rate: 1 °C min^−1^) at shear rates of 1 and 100 s^−1^.

### Western Blot and Exosome Characterization

The EV surface marker and other protein content were quantified by western blot. Cells and extracellular vesicles (Evs) were lysed using RIPA lysis buffer supplemented with protease inhibitor, and the lysates were incubated on ice for 30 min. Proteins were then denatured at 95 °C for 10 min. Each protein sample was separated into an SDS‐PAGE gel and transferred to a 0.45 µm nitrocellulose membrane (Invitrogen, Cat# STM2008). The membranes were blocked with 5% BSA for 1 h at room temperature and incubated with primary antibodies overnight at 4 °C. The primary antibodies used were as follows: β‐actin mouse antibody (CST, Cat# 3700s, 1:1000), CD81 rabbit antibody (CST, Cat# 56039S, 1:1000), HIF‐1α (CST, Cat# 14179S, 1:1000), and VEGF‐A (CST, Cat# 50 661, 1:1000). HRP‐linked secondary antibodies, anti‐mouse IgG (CST, Cat# 7076S, 1:1000) and anti‐rabbit IgG (CST, Cat# 7074S, 1:1000), were applied for 1 h at room temperature. Immunoblots were visualized using enhanced chemiluminescence (ECL) substrate (Thermo Fisher Scientific, Cat# 34 580) and imaged by an iBright FL1000 imaging system (Thermo Fisher Scientific). Total protein was quantified using ImageJ software.

Quantitative RT‐PCR Assay was employed to quantify miRNAs. EV miRNAs were extracted using TRIzol reagent (Invitrogen, Cat# 15 596 026) according to the manufacturer's instructions. Total RNA was quantified, and the expression level of miRNA 9 was evaluated using the miRCURY LNA miRNA PCR Starter Kit (QIAGEN, Cat# 339 320). Quantitative RT‐PCR was performed on the StepOnePlus real‐time PCR system (Applied Biosystems). miRNA 103 served as an internal control. Relative expression levels were calculated using the 2–ΔΔCt method.

### Untargeted Metabolomics to Identify Small Molecule Metabolites

For lipid membrane lysis, 10 µL of the isolated EV suspension was added to 1 mL of degassed methanol. The lysate was then analyzed via liquid chromatography‐mass spectrometry (LC‐MS) using an Xevo G2‐XS Qtof instrument, following previously established protocols.^[^ ^24^ ^]^ Chromatographic data were processed and analyzed using MetaboAnalyst.

### In Vitro Validation of the EV's Neurogenic Capability

EVs were isolated from control or DFO‐conditioned iPSC‐NPCs. The EVs were then incubated with iPSC‐NPCs starting on Day 1 after differentiation. The media with EVs was re‐administrated every 2 days until day 14, in which the cells were fixed and immunostained with TUJ1 and MAP2. Measurements corresponding to the secondary antibody were taken on a Tecan microplate reader.

### Animal Procedures and Surgical Protocol

All animal procedures were conducted at CHA University Laboratory Animal Center following protocols approved by the Institutional Animal Care and Use Committee (IACUC‐220189). Female Sprague–Dawley rats (10 weeks old, 230 ± 30 g; Koatech, Pyeongtaek, Korea) were maintained under controlled conditions (55–65% humidity, 24 ± 3 °C, 12‐h light/dark cycle) with one week of acclimatization before experimentation.

Animals were anesthetized with zolazepam/tiletamine (Zoletil, 50 mg kg^−1^, i.p.; Virbac Laboratories, France) and xylazine (Rompun, 10 mg kg^−1^, i.p.; Bayer, Korea). Following hair removal and skin disinfection with iodine and 70% ethanol, a craniotomy (5 mm diameter) was performed between Bregma and Lambda using a 1‐mm diamond burr (Strong 207A; SAESHIN PRECISION).

TBI was induced using a CCI device (68099II; RWD Life Science) with the following parameters: 4.0 m s^−1^ velocity, 3.0 mm depth, 5.0 s dwell time, and 5 mm diameter impact tip. Following hemostasis, treatment groups received BIOGEL alone, control EV‐loaded BIOGEL, or DFO‐conditioned EV‐loaded BIOGEL at the lesion site. The skin incision was sutured, and animals were maintained on a heating pad during recovery. Post‐operative care consisted of administering ketoprofen (1 mg kg^−1^) and cefazolin (15 mg kg^−1^) in 0.9% saline for three consecutive days. For subacute intervention, materials were delivered to the lesion epicenter at 7 days following CCI‐induced TBI. All experimental parameters and surgical procedures were identical to those described for acute‐phase studies. Results from this investigation are detailed in (Figures S7–S10, Supporting Information).

The experimental groups were categorized as follows: 1) Sham (n = 18), 2) TBI alone (n = 18), 3) BIOGEL alone (n = 15), 4) BIOGEL with control EVs (Control; n = 16), and 5) BIOGEL with DFO‐conditioned EVs (DFO; n = 16).

### Functional Recovery Assessments

Neurological function was evaluated using the modified neurological severity score (mNSS), a comprehensive behavioral assessment tool designed for animal models of brain injury. The mNSS system integrates assessments of sensory function, motor skills, reflexes, and balance. Evaluations were conducted at multiple post‐TBI time points—specifically on days 7, 14, 21, and 28. To ensure consistency and reliability, each evaluation was performed three times, following established protocols. The mNSS scoring system categorizes injury severity as mild (score 1–6), moderate (score 7–12), or severe (score 13–18). To maintain objectivity, all assessments were carried out by evaluators blinded to the experimental groups.

Motor function recovery was assessed using the Rotarod test, a widely recognized method for evaluating motor coordination and balance in rodent models. All animals were acclimated to the Rotarod apparatus over three days prior to TBI induction to establish a standardized baseline performance. The Rotarod test (Scitech Korea, Cat# SK‐RO02MR) was conducted pre‐injury and at post‐injury days 1, 7, 14, 21, and 28. During testing, the Rotarod apparatus was programmed to increase rotational speed progressively, from 4 rpm to a maximum of 40 rpm, with each session lasting up to 300 s.

### Enzyme‐Linked Immunosorbent Assay (ELISA)

On day 28 post‐TBI, prior to euthanasia, blood serum and cerebrospinal fluid (CSF) samples were carefully collected for analysis. CSF was obtained from the cisterna magna, a subarachnoid space at the base of the skull. The procedure involved gently flexing the animal's neck to expose the collection site, followed by precise insertion of a syringe to draw the clear CSF. The collected CSF was centrifuged at 3000 rpm at 4 °C for 10 min, and the supernatant was stored at −80 °C for preservation.

Blood samples were drawn from the dorsal aorta via laparotomy, performed prior to perfusion. The blood was immediately transferred into K2EDTA‐coated tubes (BD Microtainer, Cat# 365 974) to prevent coagulation. Samples were centrifuged at 3000 rpm at room temperature (23 °C) for 15 min to isolate the serum. The resulting supernatant was carefully collected and stored at −80 °C for subsequent analysis.

Quantitative biochemical analyses were performed using enzyme‐linked immunosorbent assay (ELISA). BDNF levels in CSF were measured with the Total BDNF Quantikine ELISA Kit (R&D Systems, Cat# DBNT00). Interleukin‐10 (IL‐10) concentrations in blood serum were assessed using the IL‐10 ELISA Kit (Abcam, Cat# ab214566). All assays were conducted in strict compliance with the protocols provided by the respective manufacturers.

### Tissue Processing and Paraffin Sectioning for Histological Analysis

For histological analysis and immunostaining, brain tissue samples were harvested on day 28 post‐TBI. The perfusion process commenced with the transcardial infusion of over 100 mL of saline to clear circulating blood, followed by fixation using 50 mL of 4% paraformaldehyde (BIOSESANG, Cat# P2031). After perfusion, the skull was carefully removed, and the brain was extracted and immersed in 4% paraformaldehyde for an additional 48 h to ensure thorough fixation.

The fixed brain tissue underwent a graded dehydration protocol, involving sequential immersion in ethanol solutions of increasing concentrations: 70%, 80%, 90%, 95%, and 99.9%. This was followed by tissue clearing using a series of xylene solutions, starting with 50% xylene in 99.9% ethanol, progressing to 66.6% xylene in ethanol, and concluding with pure 99.9% xylene (Samchun, Cat# X0019).

Post‐dehydration, the tissue was embedded in fresh paraffin wax through two consecutive immersions, each lasting one hour. The paraffin‐embedded tissues were sectioned into 5‐µm slices using a microtome (Leica, Cat# RM2255). The sections were rehydrated by immersion in 40% ethanol, warmed, and floated in distilled water maintained at 48 °C to facilitate uniform expansion. Finally, the sections were carefully mounted onto glass slides. All tissue sections were prepared in the coronal plane to ensure the inclusion of the lesion site in each sample.

### Luxol Fast Blue Staining

Luxol Fast Blue staining was employed to assess neurons and myelination in brain tissue sections. The procedure was performed on 5‐µm‐thick sections obtained from paraffin‐embedded brain tissues using the Luxol Fast Blue Staining Kit (Abcam, Cat# ab150675). Sections were first deparaffinized by immersion in xylene (99.9%) and a graded ethanol series (99.9%, 95%, 90%, 80%, and 70%), followed by washing in distilled water.

The sections were stained with Luxol Fast Blue solution at a controlled room temperature of 25 ± 2 °C for 24 h. After staining, the sections were rinsed with distilled water and differentiated in 0.05% lithium carbonate solution for 20 s, followed by a brief immersion in 70% ethanol. To enhance cytoplasmic visualization, Cresyl echt violet staining was applied for 3 min. Subsequently, the sections were cleared twice in 99.9% xylene for 10 min each and mounted with Canada balsam (Reagent Duksan, Cat# D1242) under a cover glass. Imaging of the stained slides was conducted using a Zeiss Axio Scan Z1 digital slide scanner to ensure high‐resolution capture of histological details.

### Hematoxylin and Eosin Staining for Tissue Morphology

Hematoxylin and Eosin (H and E) staining was performed to evaluate tissue morphology. Unstained slide samples were first deparaffinized in xylene (99.9%) through three 10‐min intervals and then rehydrated via a descending ethanol series: 99.9% ethanol (two washes, 5 min each), followed by 95%, 90%, 80%, and 70% ethanol (3 min each). Rehydrated slides were rinsed under running tap water for 3 min.

Nuclear staining was achieved using Harris Hematoxylin (BBC Biochemical, Cat# 3550) for 10 min, followed by a 3‐min rinse under running tap water. Decolorization was performed using 1% Alcoholic HCl (1% HCl in 70% ethanol), achieved by three brief immersions and followed by another 3‐min rinse under running tap water. Neutralization was conducted using 1% ammonia water (prepared in distilled water) for 1 min, with an additional 3‐min rinse under running tap water.

Cytoplasmic staining was carried out using Eosin Y Alcoholic (BBC Biochemical, Cat# 3610) for 1 min. The slides were then dehydrated through an ascending ethanol series (70%, 80%, 90%, and 95%) and two sequential immersions in 99.9% ethanol (1 and 3 min, respectively). Clearing was performed using 99.9% xylene with two 10‐min intervals.

Finally, the stained slides were mounted using Canada balsam (Reagent Duksan, Cat# D1242) under a cover glass. High‐resolution imaging of all H and E‐stained slides was performed using a Zeiss Axio Scan Z1 digital slide scanner to capture detailed morphological features.

### Immunofluorescence Analysis

Immunofluorescence analysis was performed to visualize specific antigens in the tissue samples. Slide samples were deparaffinized in xylene (99.9%) and rehydrated through a graded ethanol series (99.9%, 95%, and 90%). Following rehydration, slides were washed with distilled water and subjected to antigen retrieval using pepsin (GBI Labs, Cat# E06‐50) for 10 min. Afterward, the slides were washed twice with PBS‐T (1× PBS with 0.1% Tween 20; Sigma–Aldrich, Cat# P9416‐100 ML), each wash lasting 2 min.

To block non‐specific binding, slides were incubated with a blocking solution (GBI Labs, Cat# E07‐100) for 1 h. The primary antibody (details provided in Supporting Information) was prepared in the appropriate dilution buffer (GBI Labs, Cat# E09‐300) and applied to the slides for 24 h at 4 °C. Following primary antibody incubation, the slides were washed twice with PBS‐T for 2 min each.

Detection involved incubation with a secondary antibody tailored to the host species of the primary antibody for 50 min. Post‐secondary antibody incubation, the slides were washed three times with PBS‐T (2 min per wash) and rinsed with Dulbecco's PBS (DPBS, Cytiva, Cat# SH30028.02). Nuclear counterstaining was performed using DAPI (300 mm; Invitrogen, Cat# D1306) at a 1:1000 dilution for 10 min. The slides were subsequently washed with PBS‐T and distilled water. The samples were then mounted using DAKO Mounting Medium (Invitrogen, Cat# S3023). Imaging of immunofluorescence‐stained slides was performed using a Zeiss Axio Scan Z1 digital slide scanner.

To ensure unbiased and representative quantification, three non‐overlapping regions of interest (ROIs) were randomly selected from each tissue section. Quantitative analysis of fluorescence intensity within these ROIs was conducted using Zen 3.1 (Blue Edition) software.

### Computer Graphics

Data visualizations were primarily created using Prism (GraphPad Software), unless otherwise specified. Specific graphics were generated using ChemDraw, while select illustrations were created using BioRender.com. Figure assembly and layout were completed using CorelDRAW.

### Statistical Analysis

Data obtained from the digital slide scanner (Zeiss Axio Scan Z1, Carl Zeiss) were analyzed using Zeiss Zen 3.1 Blue Edition software. Results are presented as mean ± standard error of the mean (SEM) to ensure a clear and accurate representation of findings.

Statistical analyses were primarily performed using one‐way analysis of variance (ANOVA) followed by Tukey's post‐hoc test for multiple comparisons. These analyses were conducted using GraphPad Prism software (version 8.0.2, GraphPad Software). For behavioral assessments, including mNSS scoring and Rotarod testing, two‐way ANOVA with Tukey's post‐hoc test was employed to account for repeated measures and interactions between experimental factors.

Statistical significance was determined using p‐values, with a threshold of *p* < 0.05 indicating statistical significance (95% confidence interval). For highly significant findings, a threshold of *p* < 0.0001 was used, corresponding to a 99.9% confidence interval. Specific details regarding statistical significance and the methodology of analysis are provided in the figure legends within the results section.

## Conflict of Interest

The authors declare no conflict of interest.

## Supporting information



Supporting Information

## Data Availability

Processed data and supplemental information are available online. Raw data necessary to reproduce the findings can be obtained from the corresponding authors upon reasonable request.
