# Supplementary material for: Advanced Biomaterial Delivery of Hypoxia‐Conditioned Extracellular Vesicles (EVs) as a Therapeutic Platform for Traumatic Brain Injury
Source: Adv Sci (Weinh). 2025 Sep 8;12(44):e04147. doi: 10.1002/advs.202504147 (PMC12667507; doi:10.1002/advs.202504147)
Supplement: Supplementary file 1 — Supporting Information [file ADVS-12-e04147-s001.docx]

**Supporting Information**

Advanced Biomaterial Delivery of Hypoxia-Conditioned Extracellular Vesicles (EVs) as a Therapeutic Platform for Traumatic Brain Injury

Joshua B. Stein^#^, Songzi Zhang^#^, Eun Ji Roh, Jeffrey Luo, Meizi Chen, Hyunjun Jang, Li Ling Goldston, Brandon Conklin, Inbo Han*, Ki-Bum Lee*

J. B. Stein, J. Luo, M. Chen, H. Jang, L.L. Goldston, B. Conklin, Prof. K.-B. Lee*

Department of Chemistry and Chemical Biology

Rutgers, The State University of New Jersey

Piscataway, NJ 08854, USA

E-mail: kblee@rutgers.edu

Website: <https://kblee.rutgers.edu/>

Telephone: (848) 445-2081

Fax: (732) 445-5312

S. Zhang, E. J. Roh, Prof. I. Han*

Department of Neurosurgery

CHA University School of Medicine, CHA Bundang Medical Center

59 Yaptap-ro, Bundang-gu, Seongnam-si, Gyeonggi-do 13496, Republic of Korea

E-mail: hanib@cha.ac.kr

Website: <https://sites.google.com/view/inbolab/home>

Telephone: +82 31-780-1924

Fax: +82 31-780-5929

# J. B. Stein and S. Zhang contributed equally to this work.

**Keywords:** Traumatic Brain Injury (TBI), Human Induced Pluripotent Stem Cell Neural Progenitor Cell (hiPSC-NPC), Extracellular Vesicles (EVs), Hypoxia Conditioning, Bioorthogonal Hydrogel (BIOGEL), Neurogenesis, Angiogenesis, Neural Repair

**
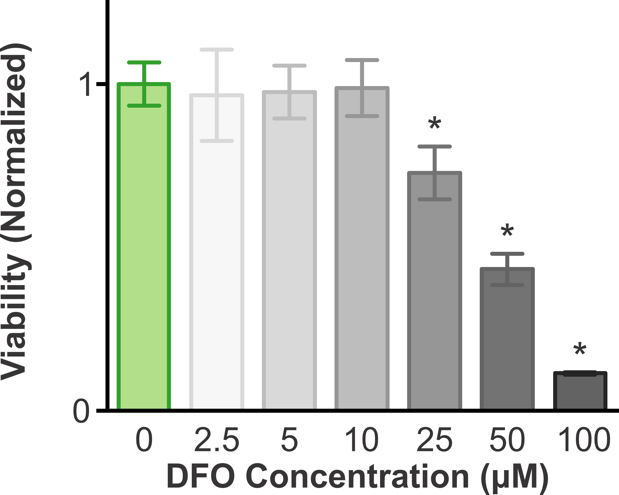
**

**Figure S1. | Effect of deferoxamine** **on hiPSC-NPC Viability at Varying Concentrations.** Human-induced pluripotent stem cell-derived neural progenitor cells (hiPSC-NPCs) were incubated with varying concentrations of deferoxamine (DFO), and cell viability was assessed using the PrestoBlue Cell Viability Assay. No significant changes in cell viability were observed at the concentration used for extracellular vesicles (EVs) harvesting (10 uM).

# **Figure S1. Change in Exosome Concentration and Size During Storage**


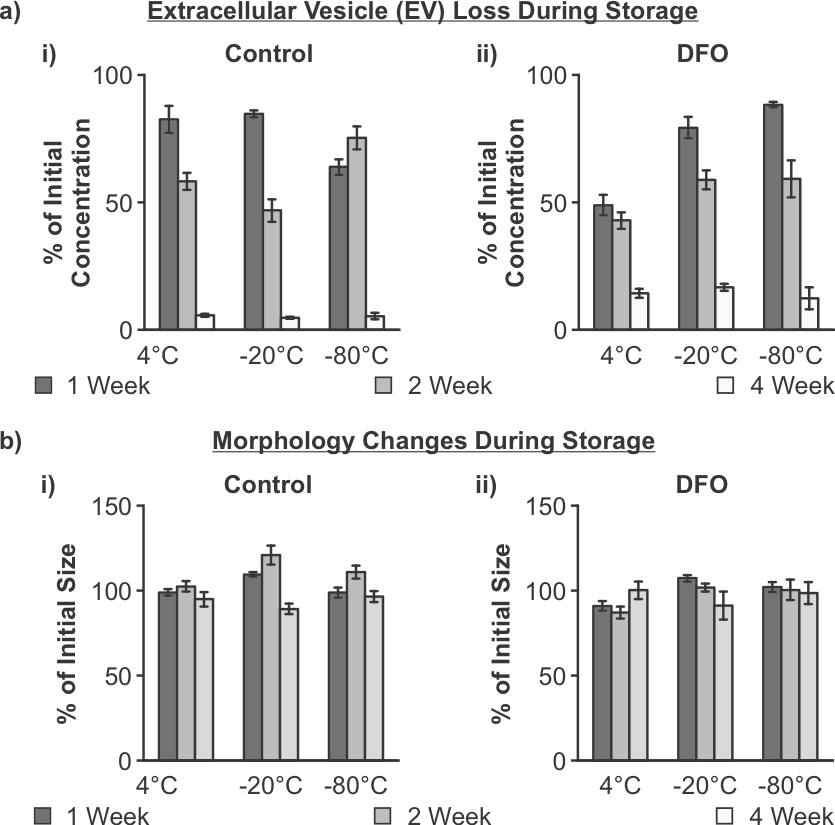


**Figure S2. | Changes in Extracellular Vesicle (EV) Concentration and Size During Storage.** Extracellular Vesicles (EV) were harvested from human NPCs. Size and concentration were measured using nanoparticle tracking analysis after storage at the indicated temperatures and time points. A significant reduction in exosome concentration was observed over time, whereas nanoparticle morphology remained unchanged.

**
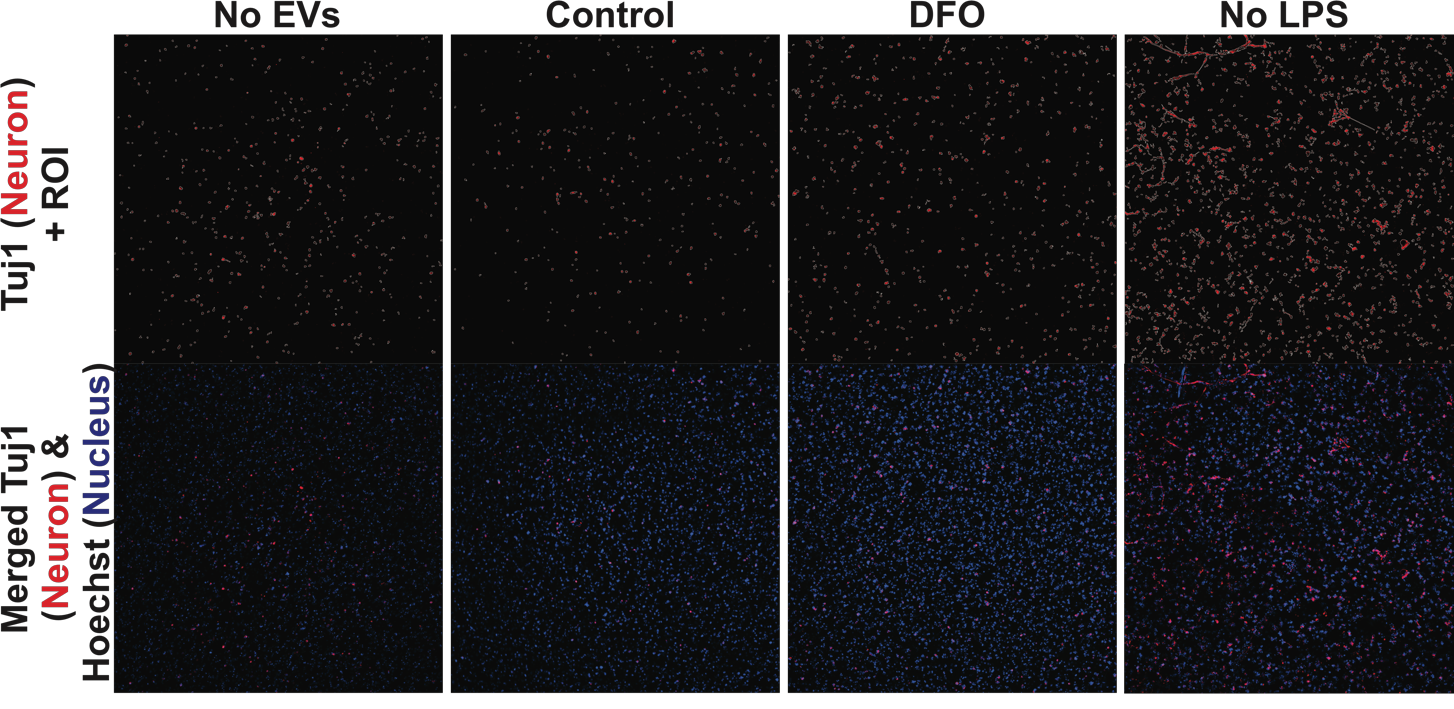
**

**Figure S3. | DFO-conditioned Extracellular Vesicle-loaded BIOGEL Promotes Neuronal Survival Under Inflammatory Conditions in vitro.** Human-induced pluripotent stem cell-derived neural progenitor cells (hiPSC-NPCs)co-encapsulated with DFO-conditioned extracellular vesicles within BIOGEL exhibited enhanced expression of the neuronal marker β-III tubulin (TUJ1) when exposed to neuroinflammatory stimuli.

**
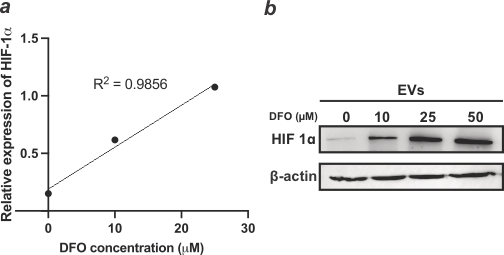
**

**Figure S4. | DFO Stabilizes the HIF-1α Complex in iPSC-NPCs. Extracellular vesicles** derived from hNPCs contained potent wound-healing factors due to the upregulation of HIF-1 **α**. The expression of HIF-1**α**  was upregulated in a concentration-dependent manner following treatment with deferoxamine (DFO) (a,b).

**
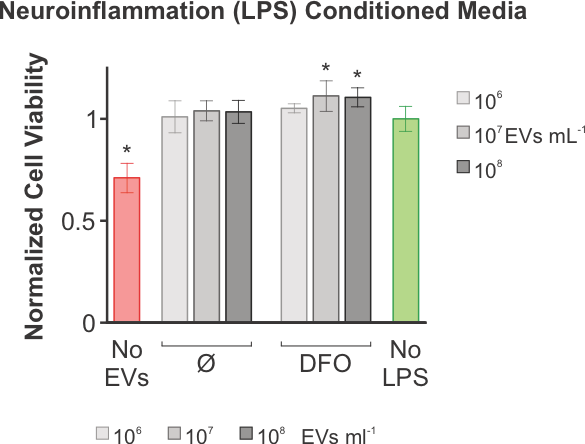
**

**Figure S5. | Cell Viability Under Neuroinflammatory Conditions after Treatment with iPSC-NPC-Derived Extracellular Vesicles.** Exposure to neuroinflammatory environments induced by lipopolysaccharide (LPS) resulted in decreased cell viability. In contrast, cell viability was significantly restored by treatment with control or DFO-conditioned extracellular vesicles (EVs) originating from hiPSC-NPCs.


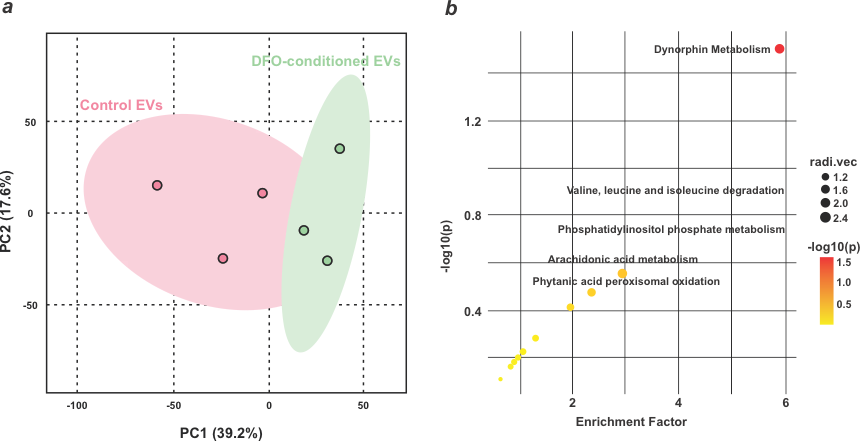


**Figure S6. | PCA Scores Plot of Control-EVs and DFO-conditioned EVs in positive mode.**


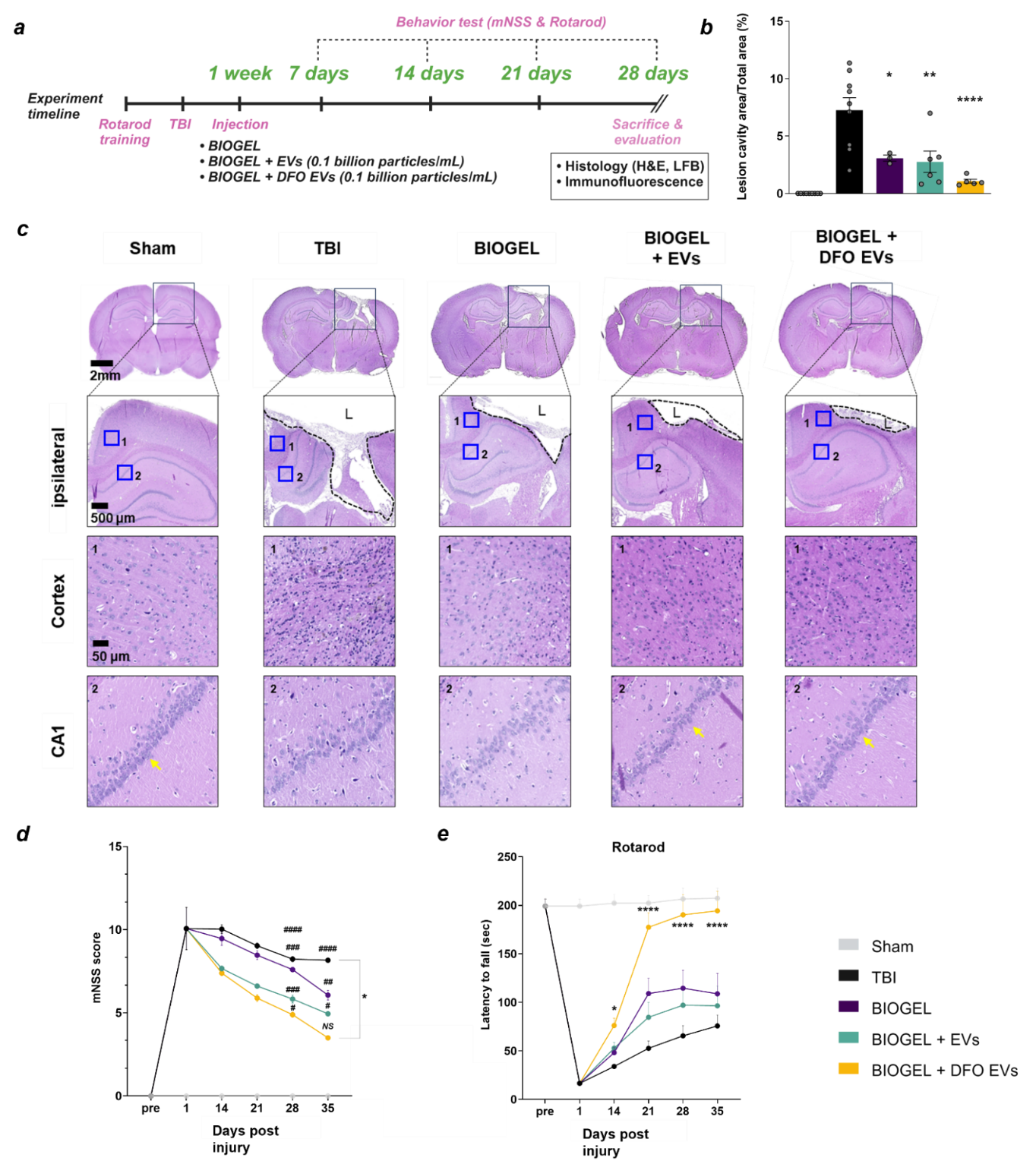


**Figure S7. | DFO-conditioned EV-loaded BIOGEL Promotes Neural Recovery in Delayed Treatment Post-TBI. (a)** Experimental timeline depicting delayed intervention protocol: BIOGEL administration at day 7 post-TBI (0.1 × 10^9 particles/mL EV concentration), behavioral assessments (mNSS and Rotarod), and endpoint analyses on day 28 for histological and immunofluorescence evaluation. **(b)** Lesion volume quantification on day 28 post-injury, expressed as a percentage of total brain volume, based on H&E staining across experimental groups. **(c)** Representative H&E-stained brain sections showing ipsilateral hemisphere, cortex, and hippocampal CA1 regions. Yellow boxes indicate magnified regions of magnification; black arrows highlight areas of reduced tissue damage in the DFO-conditioned EV-loaded BIOGEL group. **(d)** Longitudinal assessment of modified Neurological Severity Score (mNSS) showing significant functional improvement in the DFO-conditioned EV-loaded BIOGEL group compared to untreated TBI and control groups (Two-way ANOVA with Bonferroni's post hoc test). **(e)** Rotarod assessment demonstrating enhanced motor recovery in DFO-conditioned EV-loaded BIOGEL group versus untreated TBI and other treatment groups. *p < 0.05, **p < 0.01, ****p < 0.0001, NS: not significant compared to TBI group; one-way ANOVA with Tukey's post hoc test. Data are presented as mean ± SEM.


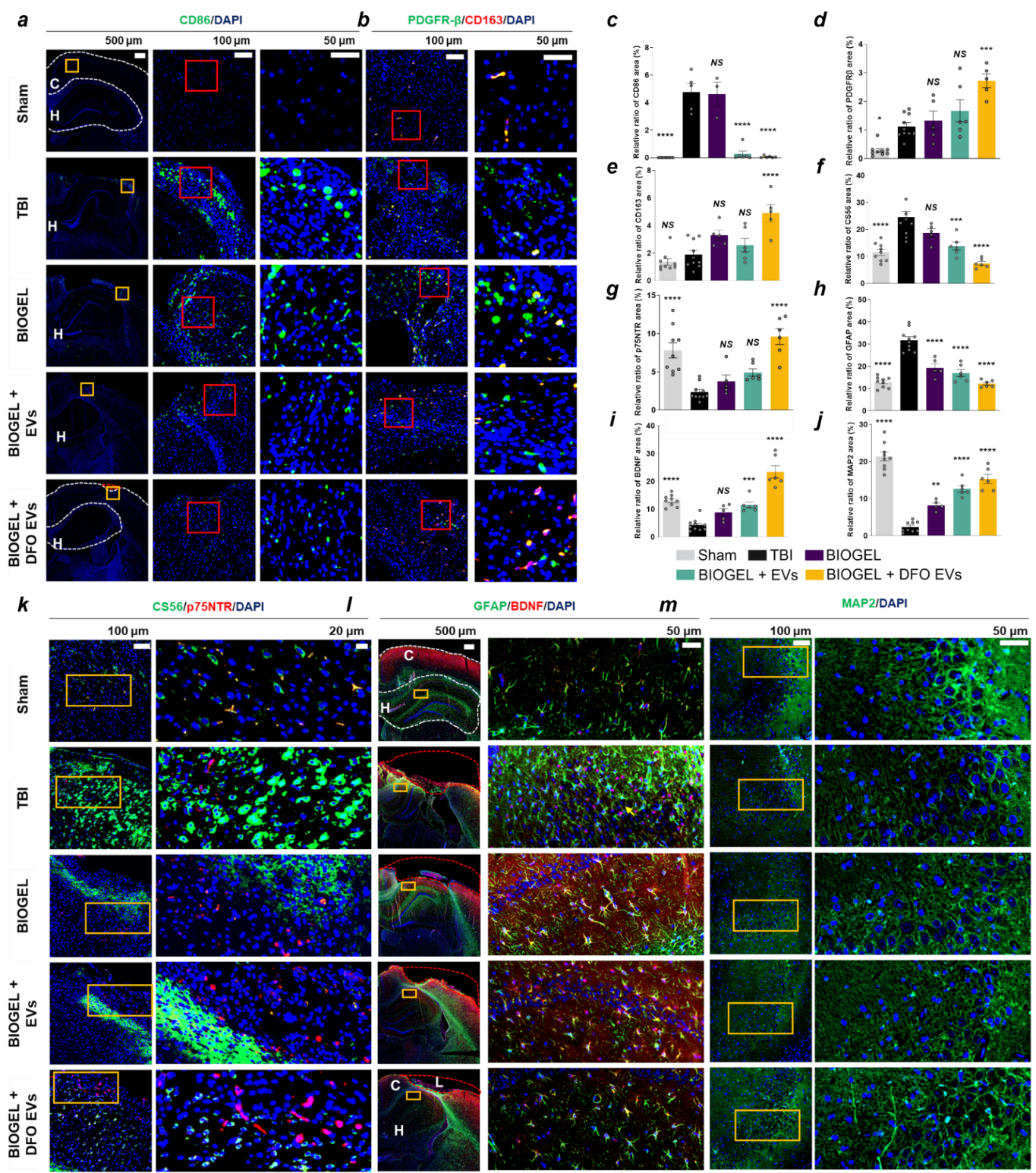


**Figure S8. | DFO-conditioned EV-loaded BIOGEL Modulates Neuroinflammation and ECM Remodeling in Delayed Treatment Paradigm. (a,b)** Representative immunofluorescence images of cortical sections. **(a)** CD86 (green) with DAPI nuclear counterstain (blue) showing attenuated pro-inflammatory microglial activation post-treatment. **(b)** PDGFR-β (green) and CD163 (red) with DAPI (blue) demonstrating increased anti-inflammatory response and pericyte recruitment. **(c-j)** Quantitative analysis immunofluorescence intensity and regenerative markers: pro-inflammatory marker CD86 **(c)**, pericyte marker PDGFR-β **(d)**, anti-inflammatory marker CD163 **(e)** ECM components CS56 **(f)**, neurotrophic receptor p75NTR **(g)**, glial scar marker GFAP **(h)**, neurotrophic factor BDNF **(i)** neuronal marker MAP2 **(j)**. Treatment significantly reduced pro-inflammatory markers and glial scarring while enhancing anti-inflammatory and regenerative markers. *p < 0.05, **p < 0.01, ***p < 0.001, ****p < 0.0001, NS: not significant compared to TBI group; one-way ANOVA with Tukey's post hoc test. **(k)** Representative immunofluorescence images showing CS56 (green) and p75NTR (red) expression with DAPI (blue) at the lesion site. **(l)** Representative hippocampal immunofluorescence images showing GFAP (green) and BDNF (red) expression with DAPI (blue). C: Cortex, L: Lesion site, H: Hippocampus. **(m)** Representative cortical immunofluorescence images showing MAP2 (green) expression with DAPI (blue), indicating enhanced neuronal preservation following treatment. Data are presented as mean ± SEM.


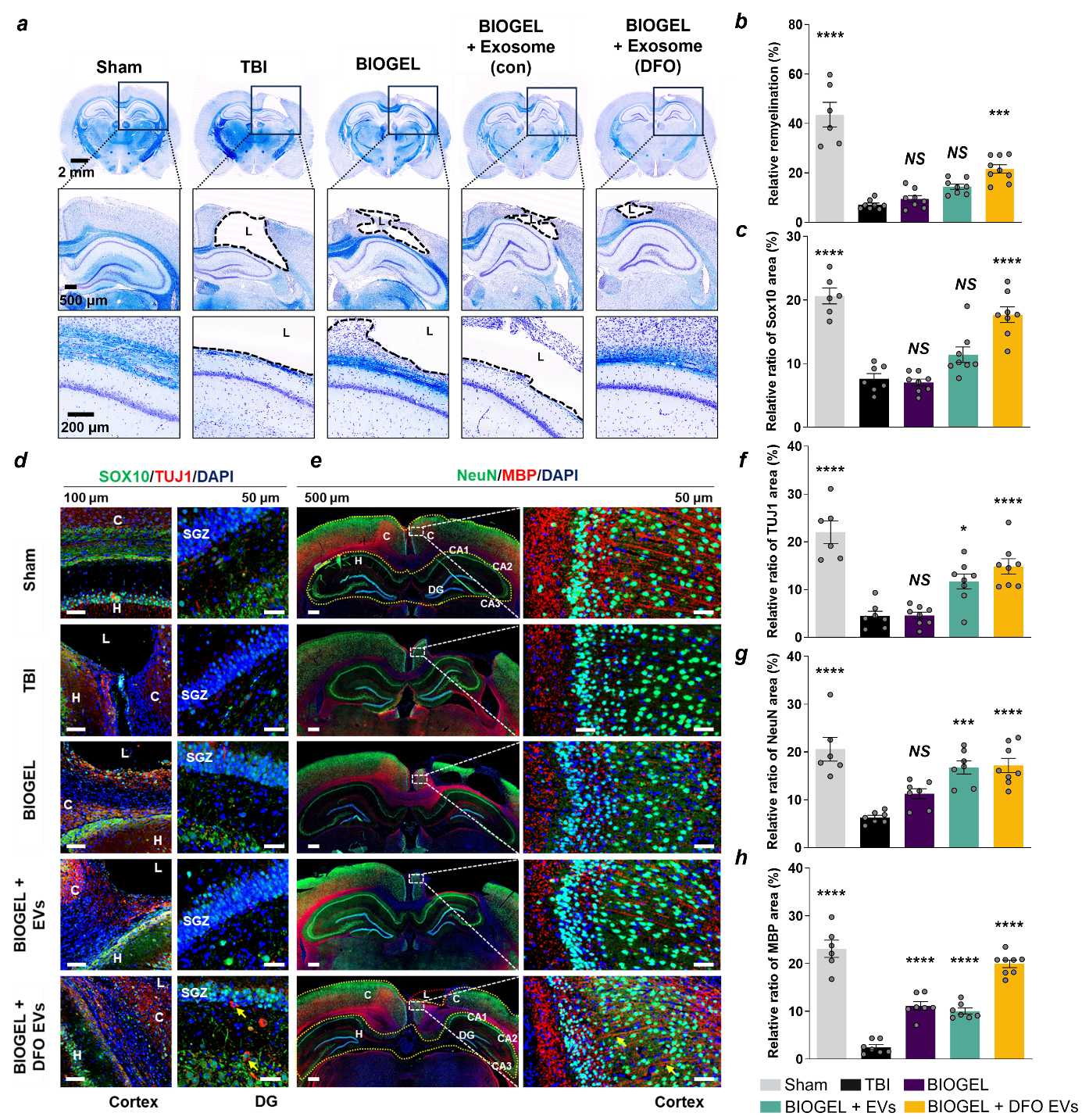


**Figure S9. DFO-conditioned EV-loaded BIOGEL promotes remyelination and neuronal maturation in cortical and hippocampal regions following TBI. (a)** Representative Luxol Fast Blue-stained brain sections showing myelin distribution across experimental groups, highlighting cortical and hippocampal regions. **(b)** Quantification of myelination across experimental groups. n = 9 (DFO-conditioned EV-loaded hydrogel), n = 8 (TBI, hydrogel alone, control EV-loaded hydrogel), n = 6 (sham). (**c)** Quantification of SOX10 immunofluorescence intensity across experimental groups. n = 8 (TBI), n = 7 (EV-loaded hydrogel groups), n = 6 (sham and hydrogel alone). (**d)** Representative immunofluorescence images showing SOX10 (green) and TUJ1 (red) expression with DAPI nuclear counterstain (blue). **(e)** Representative immunofluorescence images showing NeuN (green) and MBP (red) expression with DAPI nuclear counterstain (blue). Yellow arrowheads indicate regions of positive immunoreactivity. C: Cortex, L: Lesion site, H: Hippocampus. (**f-h)** Quantification of immunofluorescence intensity for TUJ1, NeuN, and MBP across experimental groups. n = 8 (TBI), n = 7 (EV-loaded hydrogel groups), n = 6 (sham and hydrogel alone). *p < 0.05, **p < 0.01, ***p < 0.001, ****p < 0.0001, NS: not significant versus TBI group; one-way ANOVA with Tukey's post hoc test. Data presented as mean ± SEM.

**
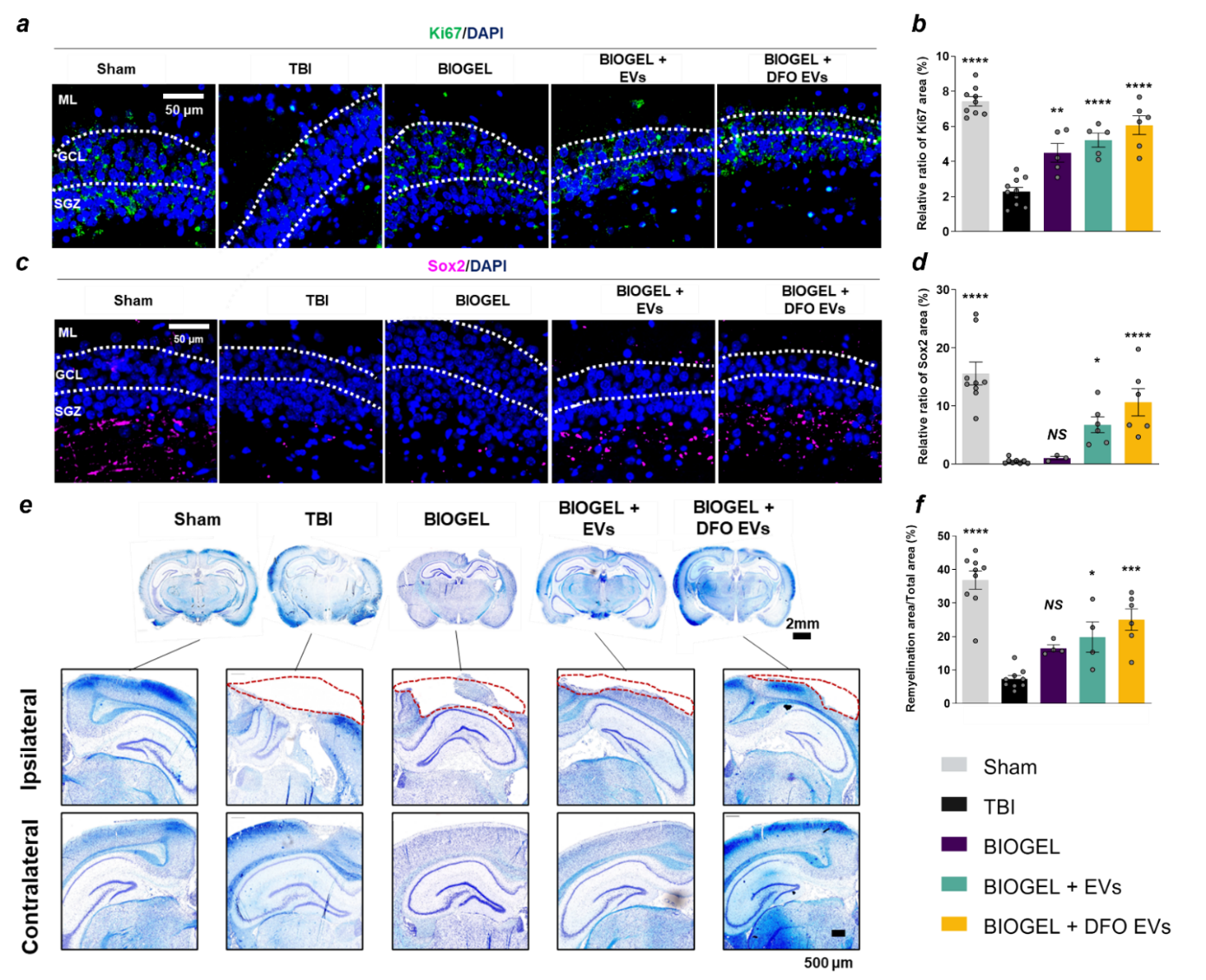
**

**Figure S10. | DFO-conditioned EV-loaded BIOGEL Enhances Neural Progenitor Proliferation and Remyelination in Delayed Treatment Paradigm.** **(a,b)** Representative immunofluorescence images of the hippocampal dentate gyrus showing Ki67-positive proliferating cells (green) and Sox2-positive neural progenitors (magenta). **(c)** Representative Luxol Fast Blue (LFB)-stained brain sections demonstrating tissue preservation and myelination patterns across treatment groups. **(d)** Quantification analysis of LFB-positive myelinated area showing enhanced myelination in the DFO-conditioned EV-laded BIOGEL group compared to other treatment groups. **(e,f)** Quantification of Ki67 and SOX2 immunofluorescence intensity across treatment groups. Data are presented as mean ± SEM. *p < 0.05; **p < 0.01; ***p < 0.001; ****p < 0.0001; NS = not significant compared to TBI group; one-way ANOVA with Tukey's post hoc test.


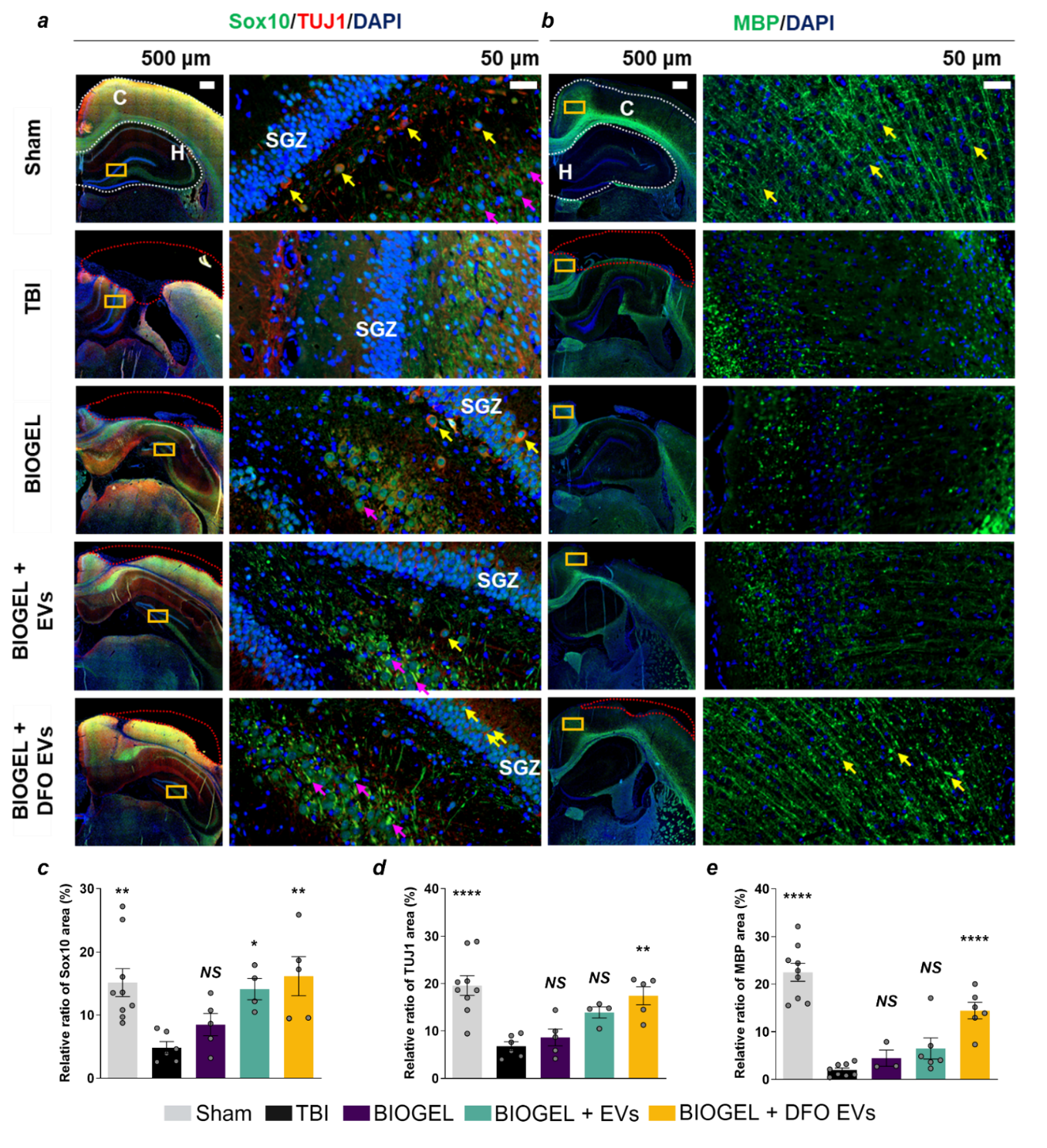


**Figure S11. | DFO-conditioned EV-loaded BIOGEL Enhances Oligodendrocyte Development and Myelination in Delayed Treatment Paradigm. (a)** Representative immunofluorescence images of hippocampal regions showing SOX10-positive oligodendrocyte lineage cells (green) and TUJ1-positive neurons (red) with DAPI nuclear counterstain (blue). Yellow arrows indicate SOX10-positive cells; magenta arrows indicate TUJ1-positive cells. **(b)** Representative cortical immunofluorescence images showing MBP (green) expression with DAPI nuclear counterstain (blue), demonstrating enhanced myelination in the treatment group. Yellow arrows indicate regions of MBP immunoreactivity in sham and DFO-conditioned EV-loaded BIOGEL groups. C: Cortex, L: Lesion site, H: Hippocampus. **(c-e)** Quantitative analysis of immunofluorescence intensity for oligodendrocyte lineage marker SOX10 **(c)**, early neuronal marker TUJ1 **(d)**, and myelin marker MBP **(e)** across experimental groups. Data are presented as mean ± SEM. *p < 0.05, **p < 0.01, ***p < 0.001, ****p < 0.0001, NS: not significant compared to TBI group; one-way ANOVA with Tukey's post hoc test.


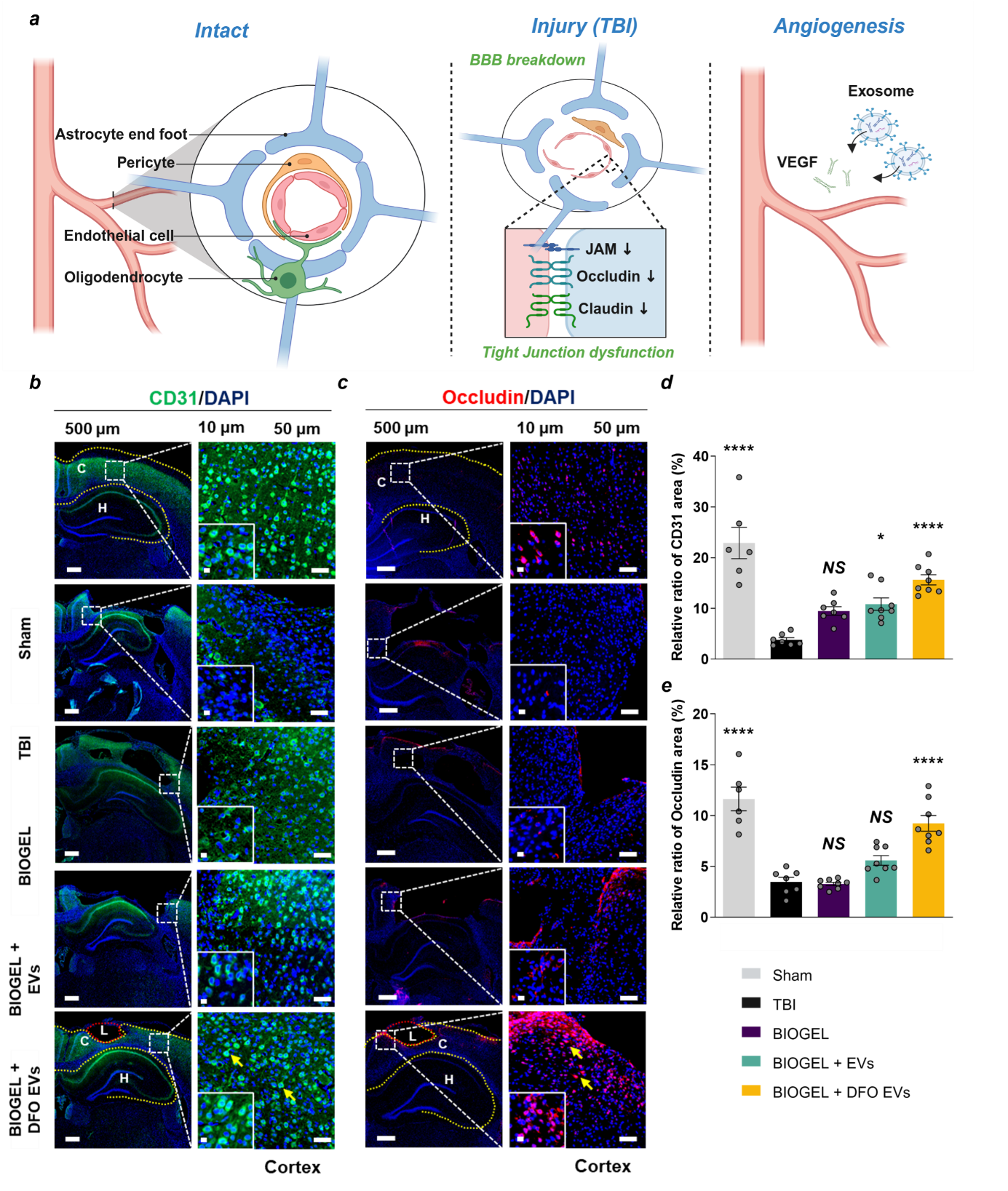


**Figure S12. DFO-conditioned EV-loaded BIOGEL promotes vascular regeneration and blood-brain barrier restoration following TBI.** a, Schematic representation of blood-brain barrier components and vascular regeneration, illustrating therapeutic mechanisms of DFO-conditioned EV treatment following TBI. b,c, Representative immunofluorescence images showing CD31 (green) (b) and Occludin (red) (c) expression with DAPI nuclear counterstain (blue) in cortical sections, demonstrating vascular integrity and tight junction formation. C: Cortex, L: Lesion site, H: Hippocampus. d,e, Quantification of immunofluorescence intensity for CD31 (d) and Occludin (e) across experimental groups. n = 8 (EV-loaded BIOGEL groups), n = 7 (TBI), n = 6 (sham and hydrogel alone). Yellow arrowheads indicate regions of positive immunoreactivity. *p < 0.05, ****p < 0.0001, NS: not significant versus TBI group; one-way ANOVA with Tukey's post hoc test. Data presented as mean ± SEM.


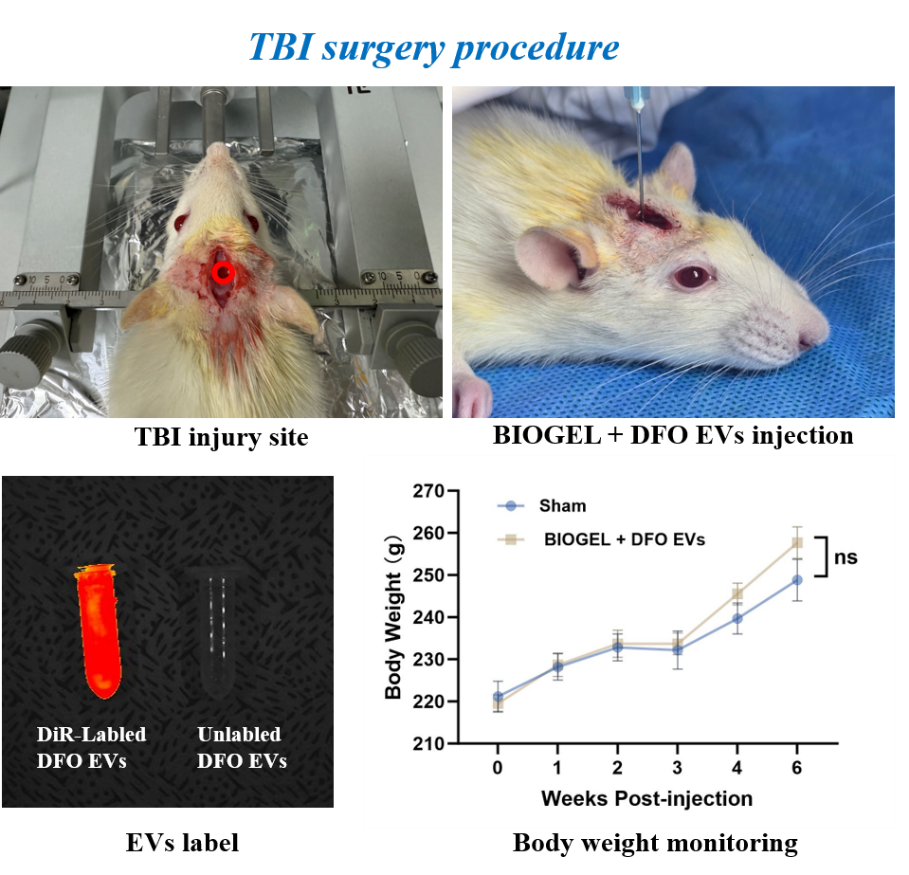


**Figure S13. . TBI Animal Surgery Procedure**


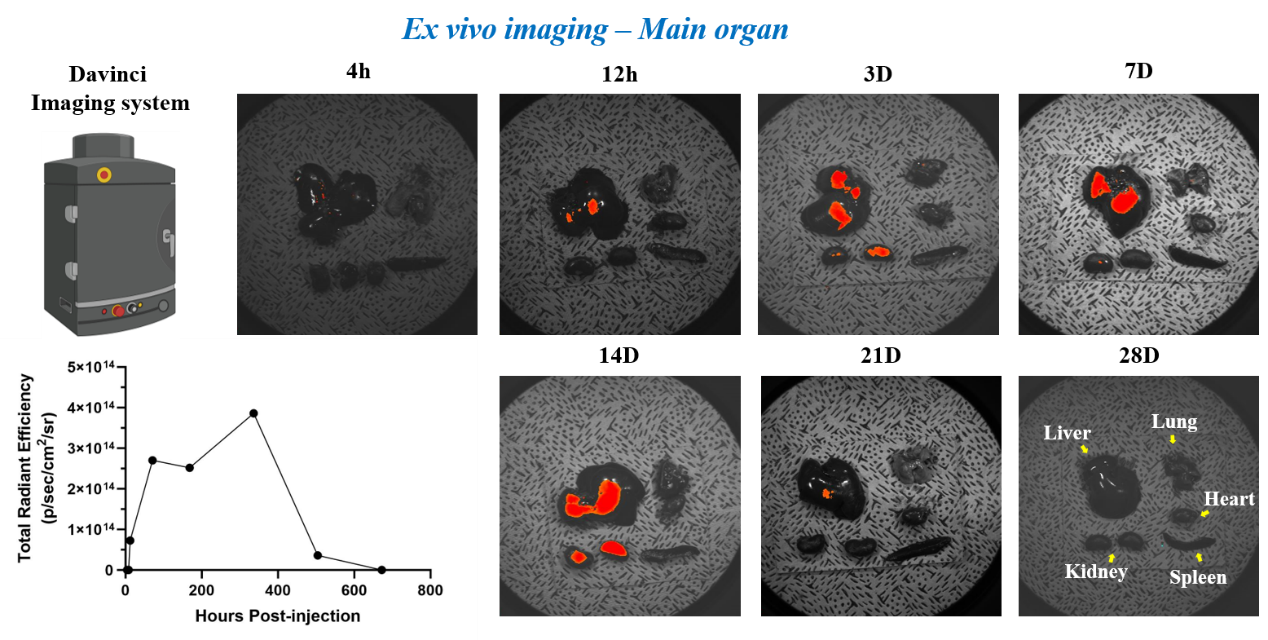


**Figure S14. In Vivo Biodistribution of DFO-EVs on the Main Organs Following Intracranial Administration.**


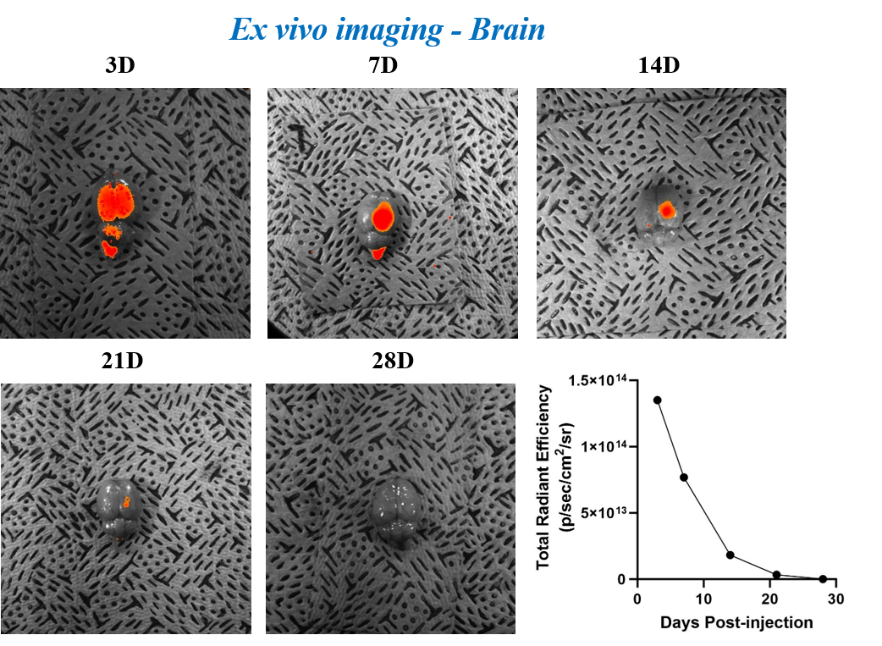


**Figure S15. In Vivo Biodistribution of DFO-EVs on the Brain Following Intracranial Administration.**

**
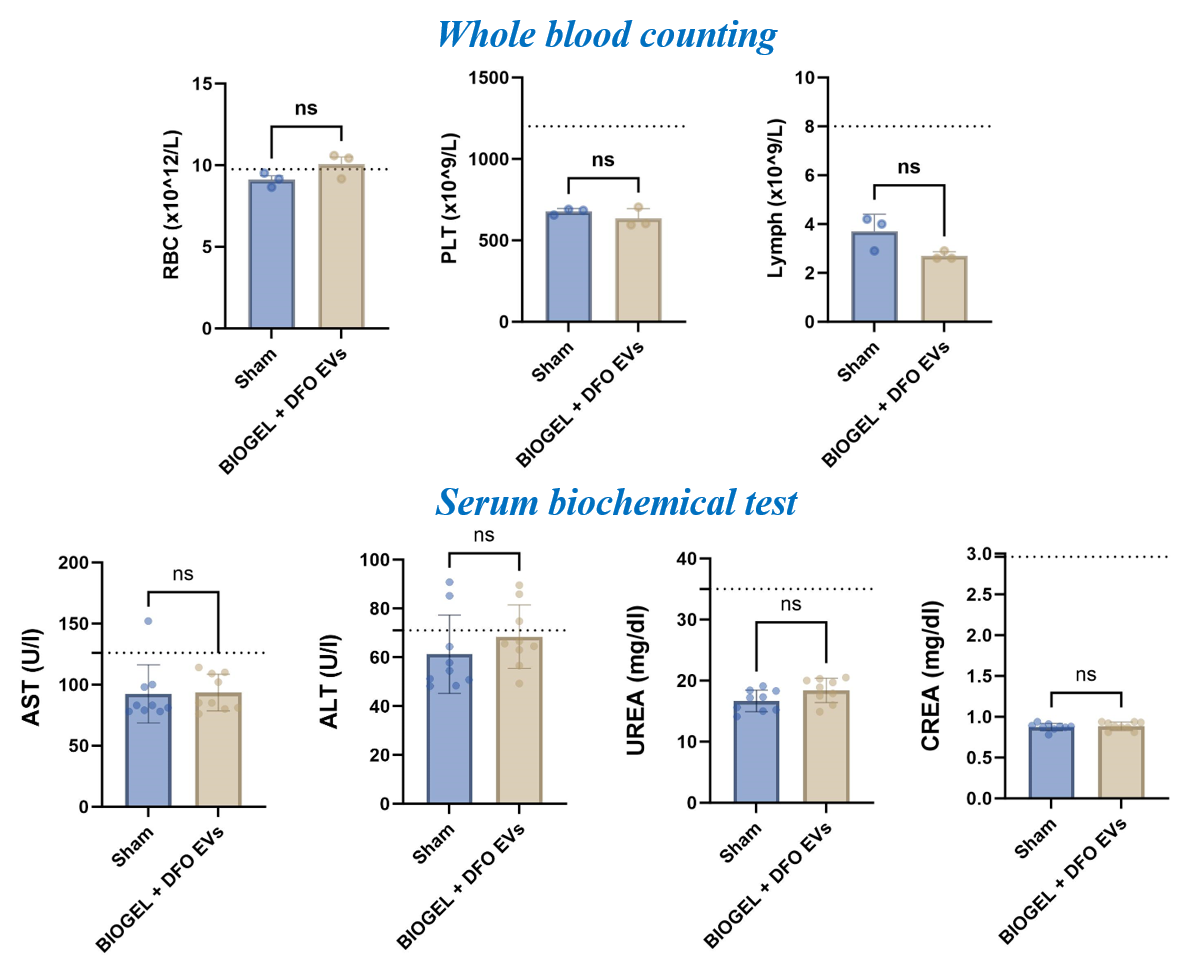
**

**Figure S16. Whole Blood Analysis Following DFO-EV-loaded BIOGEL Administration.**


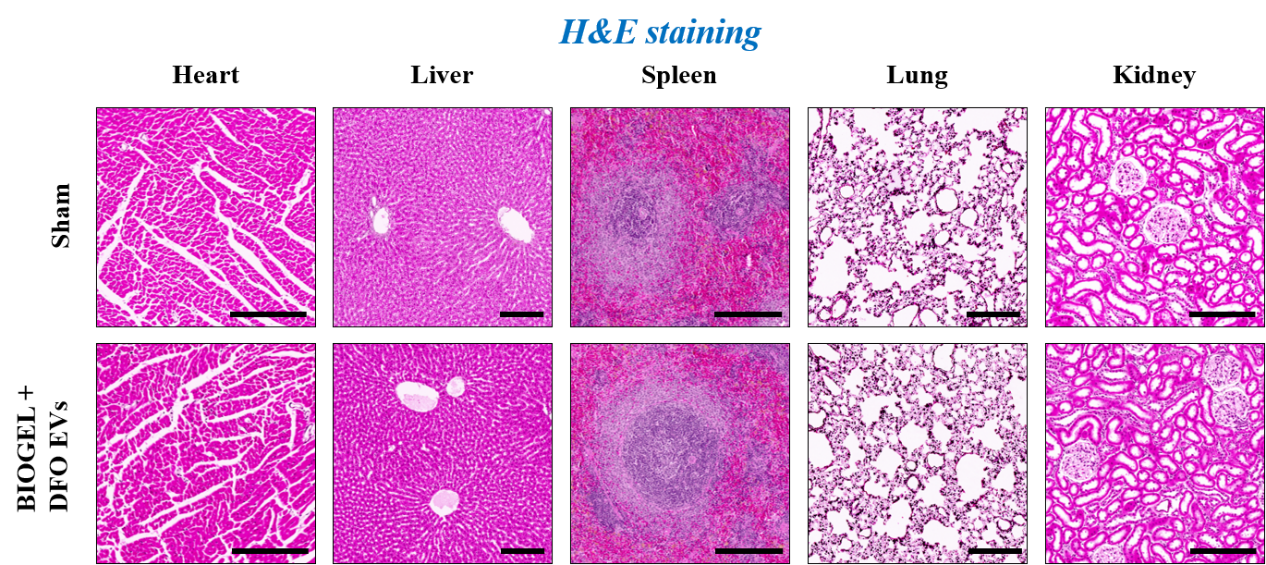


**Figure S17. Histopathological Examination Showing Intact Histological Structure and Normal Cellular Morphology Following DFO-EV-loaded BIOGEL Administration.**

| **Antigen** | **Host** | **Clonality** | **Supplier** | **Cat No.** |
| --- | --- | --- | --- | --- |
| CD86 | Mouse | Monoclonal | Abcam | Ab213044 |
| CD163 | Rabbit | Monoclonal | Abcam | Ab182422 |
| Iba-1 | Goat | Polyclonal | Abcam | Ab5076 |
| Ki67 | Rabbit | Polyclonal | Novus Biologicals | NB500-170 |
| SOX2 | Goat | Polyclonal | Novus Biologicals | AF2018 |
| GFAP | Rabbit | Polyclonal | Abcam | Ab16997 |
| BDNF | Guinea pig | Polyclonal | Alomone labs | AGP-021 |
| CS56 | Mouse | Monoclonal | Abcam | Ab11570 |
| P75NTR | Rabbit | Polyclonal | Thermo Fisher | 55014-1-AP |
| SOX10 | Mouse | Monoclonal | Novus Biologicals | MAB2864 |
| TUJ1 | Rabbit | Polyclonal | Abcam | Ab18207 |
| NeuN | Mouse | Monoclonal | Novus Biologicals | NBP1-92693 |
| MBP | Rabbit | Polyclonal | Abcam | Ab65988 |
| MAP2 | Chicken | Polyclonal | Abcam | Ab5392 |
| Syn1 | Rabbit | Polyclonal | Novus Biologicals | NB300-104-100ul |
| CD31 | Rabbit | Monoclonal | Abcam | Ab182981 |

Table 1. List of Primary Antibodies used for this study.

**THE TABLE OF CONTENTS ENTRY**

**KEYWORD**

Traumatic Brain Injury (TBI), Human Induced Pluripotent Stem Cell Neural Progenitor Cell (hiPSC-NPC), Extracellular Vesicles (EVs), Hypoxia Conditioning, Bioorthogonal Hydrogel (BIOGEL), Neurogenesis, Angiogenesis, Neural Repair

**AUTHORS**

Joshua B. Stein^#^, Songzi Zhang^#^, Eun Ji Roh, Jeffrey Luo, Meizi Chen, Hyunjun Jang, Li Ling Goldston, Brandon Conklin, Inbo Han*, Ki-Bum Lee*

Advanced Biomaterial Delivery of Hypoxia-Conditioned Extracellular Vesicles (EVs) as a Therapeutic Platform for Traumatic Brain Injury

**ToC Figure**


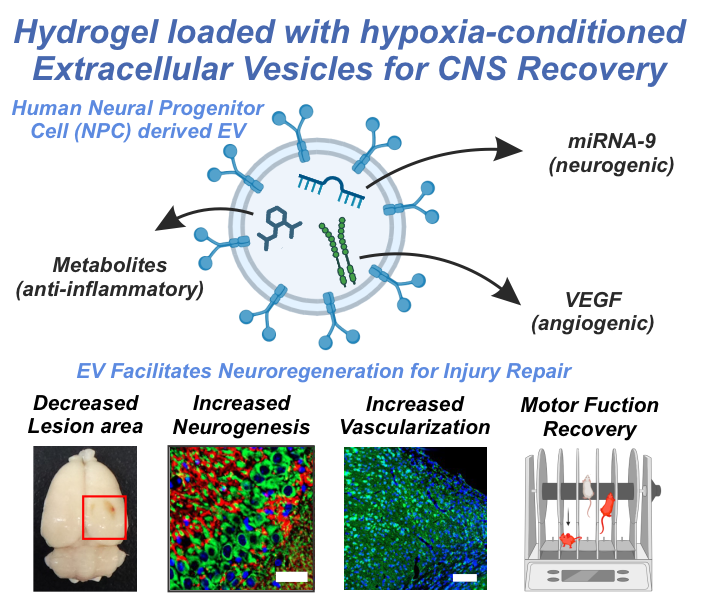


**TEXT FOR THE JOURNAL’S TABLE OF CONTENTS (TOC):** This research introduces a novel approach to enhance neuroregeneration following Traumatic Brain Injury (TBI). Extracellular Vesicles (EVs) were isolated from human neural progenitor cells under hypoxic conditions, leading to enhanced expression of neurogenic and angiogenic factors. Substantial reversal of injury pathology was observed, including increased neurogenesis, angiogenesis, NSC differentiation into neurons, and reduced inflammation.
